# Repurposing methimazole to promote coronary collateral circulation through MAPK1-mediated macrophage polarization via ferroptosis

**DOI:** 10.7150/thno.111606

**Published:** 2025-06-09

**Authors:** Ling-ping Zhu, Wei He, Ke-chuan Lin, Dan Wang, Lin-lin Wang, Shuai Li, Mei-lian Yao, Jing Chen, Mei-fang Chen, Guo-gang Zhang, Chuan-chang Li, Ling-ping Zhu, Yong-ping Bai

**Affiliations:** 1 Department of Geriatric Medicine, Xiangya Hospital, Central South University, Changsha, Hunan 410008, China.; 2 Department of Cardiovascular Medicine, Xiangya Hospital, Central South University, Changsha, Hunan 410008, China.; 3 Coronary Circulation Center, Xiangya Hospital of Central South University, Changsha, Hunan 410008, China.; 4 National Clinical Research Center for Geriatric Disorders, Xiangya Hospital, Central South University, Changsha, Hunan 410008, China.; 5 Department of Emergency Medicine, Xiangya Hospital, Central South University, Changsha, Hunan 410008, China.; 6 Department of Cardiology, The Third Xiangya Hospital, Central South University, Changsha, Hunan 410013, China.

**Keywords:** coronary collateral circulation, methimazole, ferroptosis, macrophage polarization, MAPK signaling

## Abstract

**Rationale**: Coronary collateral circulation (CCC) is essential for myocardial recovery after infarction, yet effective strategies to enhance CCC formation are scarce. In this study, we aimed to identify potential FDA-approved drugs that can promote CCC after MI injury.

**Methods**: Candidate drugs were screened through multiple analyses using cMap and public CCC-related databases. Male C57BL/6J mice underwent myocardial infarction (MI) surgery, and 3D micro-CT imaging and immunostaining for smooth muscle actin (SMA) in the watershed region of the heart were employed to evaluate CCC formation. Cardiac function was assessed through Masson's trichrome staining and cardiac ultrasonography. Macrophage polarization was analyzed using flow cytometry, qRT‒PCR, and immunostaining. Additionally, a macrophage and THP-1 cell coculture system was established to simulate the *in vivo* microenvironment, and mitochondrial morphology was assessed using electron microscopy.

**Results**: Our screen revealed that methimazole (MMI) efficiently promotes CCC formation by driving the polarization of macrophages from the proinflammatory M1-like phenotype to the proangiogenic M2-like phenotype.* In vitro*, MMI enhanced the differentiation of THP-1 cells into M2-like macrophages and increased VEGFA secretion. Mechanistically, molecular docking studies confirmed a direct interaction between MMI and MAPK1, leading to the suppression of the MAPK1/ROS axis and inhibition of ferroptosis, which facilitated M2 polarization. Furthermore, *in vivo*, honokiol (HK), a MAPK activator, reversed the effects of MMI on CCC, confirming the pivotal role of the MAPK1 pathway.

**Conclusions**: This study reveals a novel therapeutic role for MMI in promoting CCC formation following MI through the modulation of macrophage polarization via the MAPK1/ROS axis-mediated inhibition of ferroptosis. These findings highlight the potential of MMI as a strategy for enhancing cardiac repair and advancing collateral circulation therapies for ischemic heart disease.

## Introduction

Coronary heart disease (CHD) remains the leading cause of mortality worldwide, with myocardial infarction (MI) as its most severe manifestation, resulting in significant morbidity 1-3. Despite advancements in reperfusion therapies, the limited formation of coronary collateral circulation (CCC) in response to ischemia remains a critical determinant of the clinical outcomes of acute MI 4, 5. Rapid and robust CCC formation following acute infarction has been shown to dramatically reduce myocardial damage and improve recovery 6, 7. However, only a small fraction of individuals demonstrate optimal CCC after infarction, and the mechanisms driving poor collateral development remain poorly understood 8-10. This gap in knowledge provides a critical opportunity to identify therapeutic strategies that could promote CCC formation and improve the outcomes of patients with MI.

The formation of CCC is a complex and dynamic process driven by a range of cellular and molecular mechanisms 11, 12. Among these mechanisms, the polarization of macrophages from the proinflammatory M1 phenotype to the proangiogenic M2 phenotype is crucial for CCC formation 13-15. After ischemic injury, macrophages transition to the M2 phenotype, which is characterized by the secretion of anti-inflammatory cytokines, growth factors, and extracellular matrix components that promote vascular remodeling 16. Early M2 polarization plays a pivotal role in CCC by promoting the proliferation, migration, and tube formation of endothelial cells (ECs), which are essential for the early stages of CCC 14. Recent advances in immunotherapy have highlighted the potential of small-molecule compounds and new drugs that target macrophage polarization 17, 18. For example, compounds such as lenalidomide, which is used in oncology, have the ability to shift macrophage polarization toward the M1 phenotype, reducing the tumor vasculature and improving outcomes 19, 20. Similarly, in other disease contexts, targeting M2 polarization has shown promise in promoting tissue regeneration and wound healing 21, 22. These emerging therapeutic strategies underscore the broad applicability and future potential applications of small-molecule immunotherapies.

Notably, although several compounds have shown potential in modulating the macrophage phenotype, the identification of specific pharmacological agents capable of enhancing the CCC remains limited 23, 24. Moreover, most studies have focused on individual molecular targets without considering the broader, integrated molecular networks of CHD 25, 26, highlighting the critical need for a comprehensive approach that integrates multiomics data to screen for novel drugs that can effectively enhance CCC. Therefore, this study aims to fill these gaps by employing multiomics analysis combined with a Connectivity Map (CMap) drug screen to identify pharmacological agents that can enhance M2 macrophage polarization and promote CCC, providing new avenues for therapeutic intervention in acute MI.

We employed a multiomics approach combined with CMap and clinical databases to identify potential pharmacological agents that could enhance CCC 27. Through this integrated analysis, we identified methimazole (MMI), an FDA-approved drug, as a promising candidate capable of modulating macrophage polarization and promoting CCC formation. We conducted both* in vitro* and* in vivo* experiments to validate these findings. *In vitro,* MMI significantly promoted the polarization of M2 macrophages, a key process in CCC formation. *In vivo,* MMI treatment enhanced heart function and promoted CCC formation in a mouse model of MI injury. These results provide compelling evidence that MMI can facilitate CCC, underscoring its potential as a therapeutic strategy for improving cardiac repair in CHD patients.

## Methods

The expanded methods section is available in *Supplementary Materials*. The animal care and experimental protocols were approved by the Ethics Committee of Xiangya Hospital, Central South University, and adhered to the National Institutes of Health (NIH) Guidelines for the Care and Use of Laboratory Animals. All investigations involving human participants were conducted in accordance with the principles of the Declaration of Helsinki.

## Results

### Identification of MMI as a novel therapeutic modulator of CCC via multiomics analyses of CMap and the GEO database

**Figure 1:**
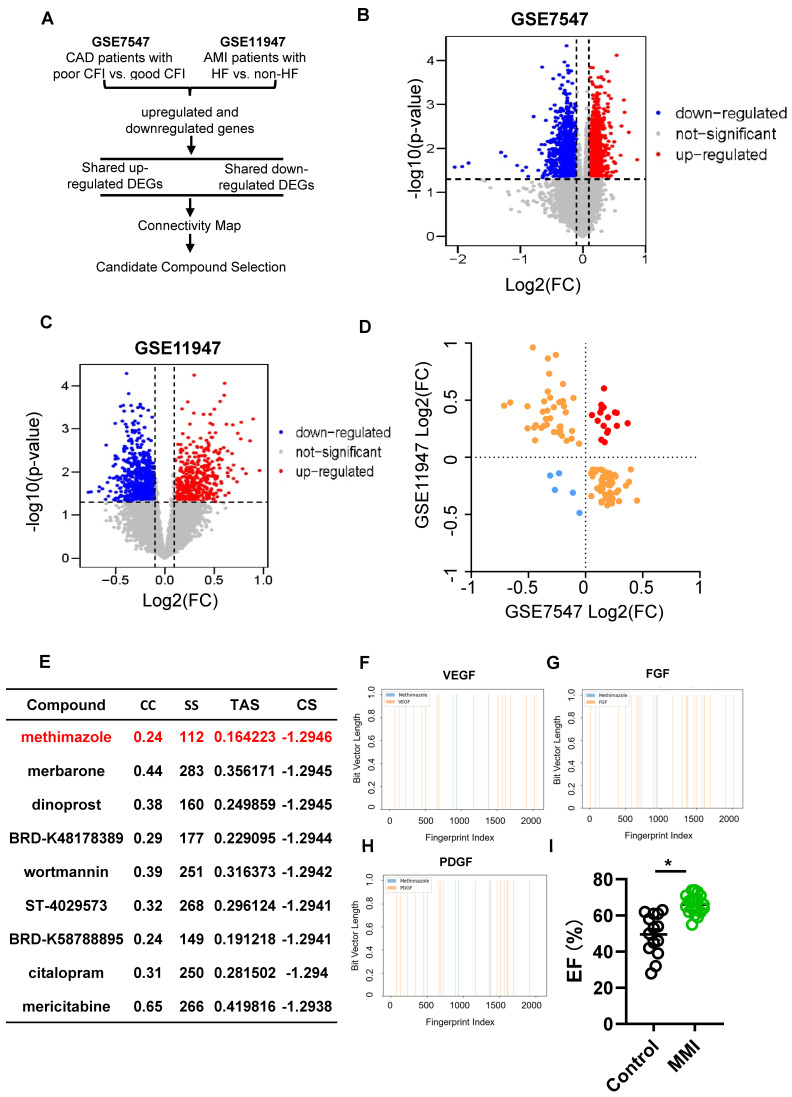
** Identification of pro-CC formation drug using CMap. A**. The flow chart of drug screening process. **B**. Differentially expressed genes (DEGs) volcano plot in dataset GSE7547 of poor collateral circulation (CC) (collateral flow index (CFI) ≤ 0.21) *vs.* good CC (CFI > 0.21). **C**. DEGs volcano plot in dataset GSE11947 of AMI patients with heart failure (HF) *vs.* non-HF. **D**. Quadrant diagram showing the DEGs distribution of GSE7547 and GSE11947, red plot represented shared upregulated genes, blue plots represented shared downregulated genes. **E**. Methimazole (MMI) was screened as the top-ranking drugs from the connectivity MAP (CMap) analysis (Connectivity Score (CS) = -0.6669). **F**. Molecular fingerprint analysis between VEGF and MMI; **G**. Molecular fingerprint analysis between FGF and MMI; **H**. Molecular fingerprint analysis between PDGF and MMI; **I**. Left ventricular ejection fraction (LVEF) of CAD patients with or without MMI treatment in our clinical cohort. Data are presented as mean ± SEM. Student's *t* test (two-tailed, unpaired) in (I) (*p <0.05).

We analyzed differentially expressed genes (DEGs) from two publicly available GEO datasets to identify therapeutic targets that promote CCC following MI injury. The first dataset (GSE7547) included human peripheral blood mononuclear cells (PBMCs) from patients with varying collateral flow indices (CFIs) and those with compared poor (CFI ≤ 0.21) and good (CFI > 0.21) CCC. The second dataset (GSE11947) compared PBMCs from patients with poor and good heart function. A schematic of the drug screening approach is shown in Figure 1A.

In total, 1,756 DEGs were identified in the GSE7547 dataset (985 upregulated and 771 downregulated), and 1,074 DEGs were identified in the GSE11947 dataset (419 upregulated and 655 downregulated) (Figure 1B-C). A quadrant analysis revealed 16 shared upregulated genes and 4 shared downregulated genes (Figure 1D). Using the CMap platform, we identified potential pharmacological agents targeting these genes, focusing on compounds with negative connectivity scores, replicate correlation coefficients > 0.3, transcriptional activity scores > 0.3, and signature strengths > 200. Among the candidates, MMI exhibited the lowest connectivity score, indicating that MMI may be most potent drug in promoting CCC (Figure 1E). Interestingly, the molecular fingerprinting analysis revealed that MMI shares structural similarities with key revascularization-related molecules such as VEGF, FGF, and PDGF at fingerprint indices of 1000 and 1500 (Figure 1F-H). These structural parallels suggest that MMI may also modulate CCC formation similarly to these growth factors. Based on these findings, MMI was selected for further investigation.

We constructed a retrospective cohort of CHD patients to validate the potential of MMI (see the characteristics in Table S1) and observed that those patients receiving MMI treatment had significantly greater left ventricular ejection fractions (LVEFs) than nonusers did (Figure 1I). These findings represent the first clinical evidence of the potential role of MMI in CCC induction, providing a novel therapeutic avenue for enhancing myocardial recovery after MI injury.

### MMI improves CCC and cardiac function in mice with MI injury

**Figure 2:**
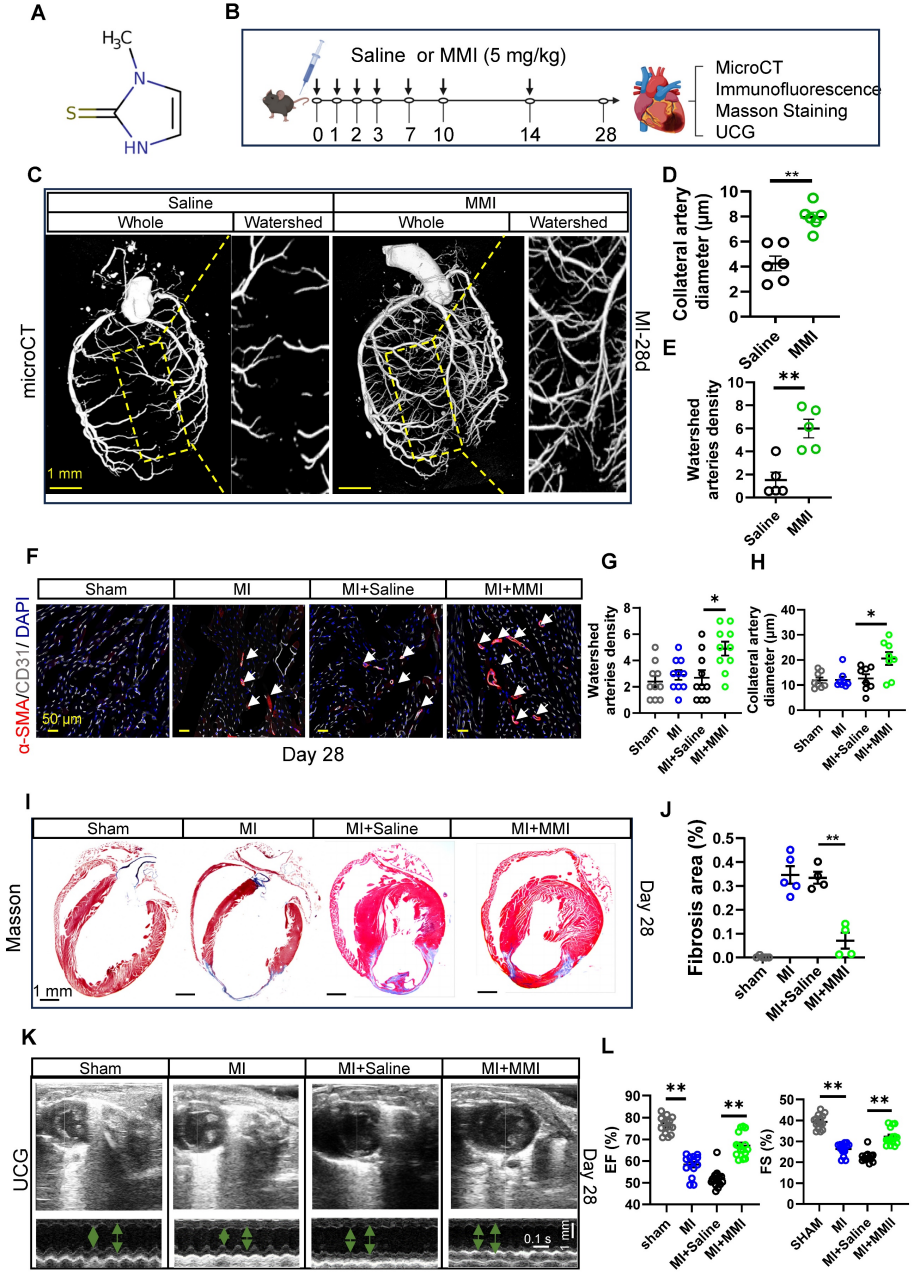
** MMI promotes CC formation and improves cardiac function in mice after myocardial infarction. A**. Simplified Molecular Input Line Entry System (SMILES) structure of the drug. **B**. Schematics of *in vivo* administration and evaluation of MMI in mice after MI. **C**-**E**. Representative microCT images **(C)** and quantification of watershed arteries diameter **(D)** and density **(E)** of reconstructed heart vasculature from mice treated with saline or MMI. Yellow area depicted watershed area between left anterior descending branch (LAD) and right coronary artery. Three-dimensional renderings (3D) images are shown (scale bar, 1 mm). **F**-**H**. Representative images of SMA (red) and CD31 (white) immunofluorescent staining **(F)** and quantification of watershed arteries diameter **(G)** and density **(H)** at 28 days after MI (white arrow indicated the collateral arteries in watershed, scale bar, 50 μm). **I** and **J** Representative images of Masson staining **(I)** and quantification of fibrosis area **(J)** at 28 days after MI (fibrosis (blue); scale bar, 1 mm). **K** and **L** Representative images of ultrasound cardiogram (UCG) to evaluate cardiac function **(K)** and quantification of EF (%) and fractional shortening FS (%) **(L)** at 28 days after MI (scale bar 1 mm, 0.1 s). One-wayANOVA with Tukey multiple comparisons test in **(G)**, **(H)**, **(J)** and **(L)** (*p<0.05, ** p<0.01, *** p<0.0001). Student's *t* test (two-tailed, unpaired) in **(D and E)** (**p<0.01). Each dot represents a single mouse **(D, E, J and L)** or a single picture **(G, H)**. Data are represented as mean ± SEM.

MMI is an imidazole-based, FDA-approved antithyroid medication that is commonly prescribed for the treatment of hyperthyroidism. While its clinical application is well established, its potential effects on CCC formation have not been reported. The chemical structure of MMI, represented by its Simplified Molecular Input Line Entry System (SMILES), is depicted in Figure 2A. Given the novel hypothesis that MMI could influence CCC, we sought to assess its efficacy in promoting CCC formation in a murine MI model.

We employed a mouse model of MI induced by permanent ligation of the left anterior descending (LAD) coronary artery, which results in profound ischemia between the LAD and right coronary artery, to investigate the effects of MMI on CCC formation. MMI (5 mg/kg) or saline (as a control) was administered intraperitoneally at the specified time points, as outlined in Figure 2B. We assessed the therapeutic efficiency of MMI for MI in the early stage by evaluating TTC staining of mouse hearts on Day 3 after MI (Figure S1A-B). We found that, compared with control mice, MMI-treated mice presented a significantly smaller necrotic area in the early stages of MI. To visualize and assess the impact on CCC, we reconstructed a three-dimensional image of the coronary artery network using micro-CT imaging to visualize and assess the impact on CCC. CCC was evaluated based on the diameter and density of arteries within the watershed area, an intermediate zone receiving blood from both the LAD and right coronary artery. At 28 days after MI, MMI-treated mice presented significantly larger arterial diameters and a greater vascular density in the watershed area than saline-treated controls did, as highlighted by the yellow boxed regions in Figure 2C-E. Immunofluorescence staining of SMA^+^CD31^+^ areas in cross-sections of the watershed area further confirmed enhanced CCC formation in MMI-treated animals (Figure 2F-H). Moreover, MMI treatment significantly reduced the fibrotic scar area, as evidenced by Masson's trichrome staining, which revealed less blue-stained fibrotic tissue in MMI-treated hearts than in control hearts at 28 days after MI (Figure 2I-J). We performed ultrasound cardiography (UCG) to assess the impact of MMI on cardiac function, and compared with saline-treated MI mice, MMI-treated mice exhibited a significant improvement in LVEF (Figure 2K-L).

We analyzed liver and kidney tissues, as well as serum samples, at 3 days post-MI to evaluate the potential side effects of MMI on organ function. Hematological assessments revealed no significant differences in red blood cell counts or hemoglobin levels between the MMI- and saline-treated groups (Figure S2A-B). The histological examination of liver and kidney tissues by H&E staining revealed no evidence of necrosis in MMI-treated mice (Figure S2C-F). Furthermore, an analysis of the serum levels of liver enzymes, including alanine aminotransferase (ALT), aspartate aminotransferase (AST), total and direct bilirubin, albumin, alkaline phosphatase (ALP), and γ-glutamyltransferase (γ-GT), revealed no significant differences between the groups (Figure S2G-N). These findings suggest that the dose of MMI used in this study does not induce adverse effects on liver or kidney function, supporting its biosecurity in the context of this preclinical model.

### MMI promotes M2 macrophage polarization at the early stage after MI injury

**Figure 3:**
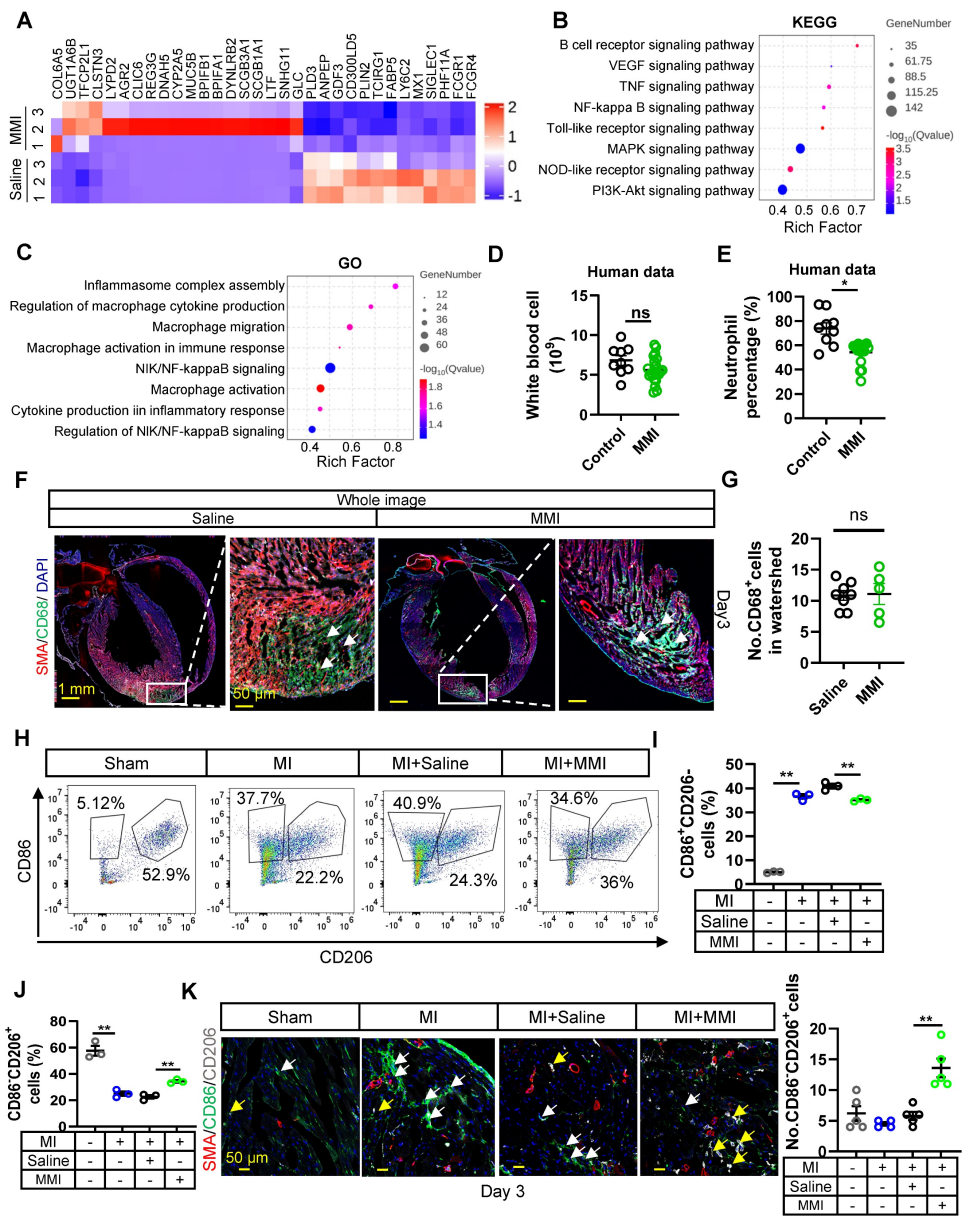
** MMI promotes M2-like macrophage polarization in MI mouse heart. A**. The heatmap showing the DEGs between heart tissue of mice treated with saline and MMI by bulk RNA seq. **B**. Kyoto Encyclopedia of Genes and Genomes (KEGG) Analysis of DEGs. **C**. Gene Ontology (GO) Analysis of DEGs. **D** and** E.** The number of white blood cell **(D)** and neutrophil percentage **(E)** in peripheral blood of CAD patients with or without MMI treatment in our cohort. **F** and **G.** The representative whole image **(F)** of SMA (red), CD68 (green) immunofluorescent staining and quantification of the CD68^+^ cells **(G)** in the watershed area at 3 days after MI (white arrow indicated the CD68+ cells in watershed area, scale bar, 1 mm and 50 μm). **H**-**J**. Flow cytometry analysis **(H)** and quantification of CD86^+^CD206^-^ (M1-like macrophage, I) and CD86^-^CD206^+^ (M2-like macrophage, J) cells rate (%).** K.** Representative images of SMA (red), CD86 (green) and CD206 (white) immunofluorescent staining (left) and quantification (right) of No.CD86^-^CD206^+^ cells (macrophage) of collaterals at 3 days after MI (the white arrow point to CD86^+^CD206^-^ cells and yellow arrow point to CD86^-^CD206^+^ cells, scale bar, 50 μm). Student's *t* test (two-tailed, unpaired) in **(D, E, G)**. One-way ANOVA with Tukey multiple comparisons test in **(I, J, K)** (ns no significance, *p<0.05, ** p<0.01, *** p<0.001). Each dot represents a single mouse **(E, I)** or a single picture **(G)**. Data are represented as mean ± SEM.

We investigated the impact of MMI on macrophage polarization during collateral circulation formation by performing bulk RNA sequencing on hearts collected from mice treated with either MMI or saline at 3 days after MI ((Figure 3A). Functional pathway analysis of DEGs using the Kyoto Encyclopedia of Genes and Genomes (KEGG) and Gene Ontology (GO) enrichment analyses revealed that the downregulated DEGs in MMI-treated mice were significantly associated with inflammatory pathways, including cytokine‒cytokine receptor interactions, TNF signaling, IL-17 signaling, and NF-kappa B signaling (Figure 3B-C). These findings suggest that MMI may exert an anti-inflammatory effect during collateral circulation formation.

In our clinical cohort, we observed a significant decrease in the leukocyte-to-neutrophil ratio in patients with coronary artery disease (CAD) who were taking MMI (Figure 3D-E). In parallel, an analysis of peripheral blood myeloid cells in a murine MI model treated with MMI revealed reductions in the numbers of neutrophils and monocytes without affecting white blood cells or lymphocytes (Figure S3A-E). These results support the hypothesis that MMI may promote collateral circulation through its anti-inflammatory actions. We examined macrophage polarization in the infarcted myocardium to further explore this mechanism. At 3 days post-MI, no significant differences in the total macrophage population (CD68^+^ cells) were observed between MMI- and saline-treated mice (Figure 3F-G). However, the flow cytometry analysis revealed a shift in macrophage polarization: while the number of M1-like (CD86^+^CD206^-^) macrophages was significantly increased in MI mice compared to sham-operated controls, MMI treatment led to a marked decrease in the number of M1-like macrophages and a corresponding increase in the number of M2-like (CD86^-^CD206^+^) macrophages (Figure 3H-J). Immunostaining further confirmed that MMI significantly increased the number of M2-like macrophages in the infarcted area (Figure 3K). Together, these findings indicate that MMI promotes a shift from a proinflammatory M1-like phenotype to an anti-inflammatory M2-like macrophage phenotype, which may contribute to improved collateral circulation after MI.

### MMI polarizes M1-like macrophages into the M2 phenotype, which triggers angiogenesis *in vitro*

**Figure 4:**
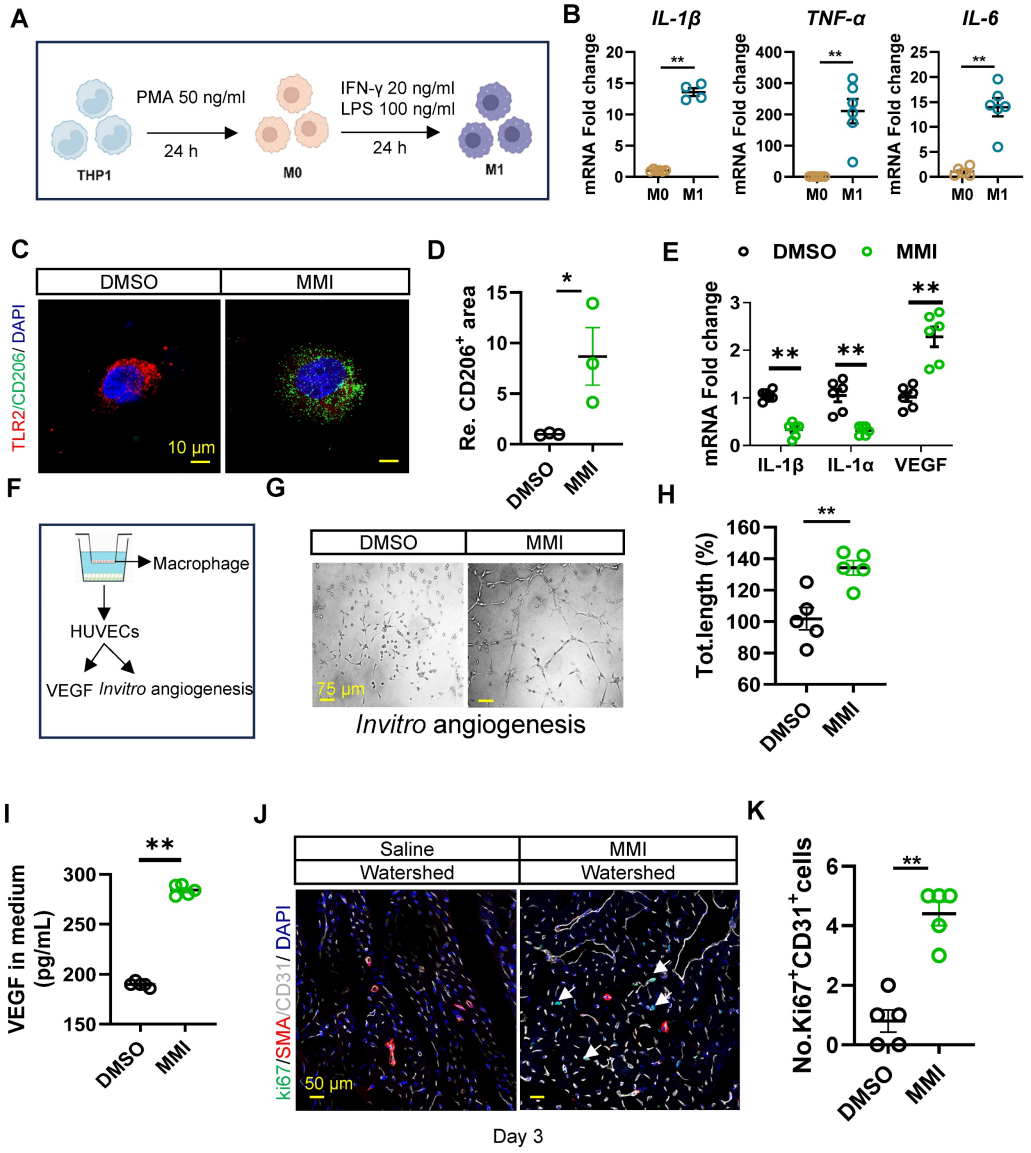
** MMI promotes M2-like macrophage polarization *in vitro*. A.** Flow chart of induced differentiation of M1-like macrophage. **B**. IL-1β, TNF-α and IL-6 mRNA fold change in M0-like and M1-like macrophage by qRT-PCR. **C** and **D.** Representative images of TLR2 (red) and CD206 (green) immunofluorescent staining **(C)** and quantification of CD206^+^ area **(D)** in M1 macrophage with DMSO or MMI treatment (scale bar, 10 μm). **E**. Quantitative RT-PCR analysis of IL-1β, IL-1α and VEGF in M2-like macrophage treated with MMI or DMSO. **F**. Schematic diagram of the co-cultured system of THP-1 cells derived macrophage and human umbilical vein endothelial cells (HUVECs). **G** and **H**. Representative images **(G)** and quantification **(H)** of *in vitro* angiogenesis of HUVECs which cocultured with DMSO or MMI treated THP-1 derived macrophage (scale bar, 75 μm),** I.** Quantification of VEGF level by ELISA in culture medium from M1 like macrophages following DMSO or MMI treatment, n = 5 independent times. **J** and** K.** Representative confocal images **(J)** and quantification **(K)** of Ki67^+^ ECs by Ki67 (green), SMA (red) and CD31 (white) immunofluorescent staining of cross section of watershed area collected from mice MI heart with saline or MMI treatment (the white arrow pointed to the Ki67^+^ endothelial cells, scale bar, 50 μm). n = 5 mice for each group. Student's *t* test (two-tailed, unpaired) in **(B, D, E, H, I and K)** (*p<0.05, **p<0.01). Data are represented as mean ± SEM.

We next examined whether MMI promotes M1-to-M2 macrophage polarization and angiogenesis *in vitro*. THP-1 cells were induced to differentiate into M0-like and M1-like macrophages to investigate this process, as shown in Figure 4A. The mRNA expression of proinflammatory cytokines, including IL-1β, TNF-α, and IL-6, was significantly increased in M1 macrophages, confirming the successful differentiation of THP-1 cells into M1-like macrophages (Figure 4B). Notably, MMI treatment induced a shift from M1-like to M2-like macrophages, as evidenced by increased immunofluorescence staining of TLR2^-^CD206^+^ cells (Figure 4C-D). Similarly, the mRNA levels of M1 markers (IL-1β and IL-1α) decreased, whereas those of M2 markers, such as VEGF, increased in MMI-treated macrophages (Figure 4E). These findings collectively demonstrate that MMI promotes M2-like macrophage polarization *in vitro*.

Since M2-like macrophages are known to secrete VEGF to stimulate angiogenesis, we further investigated whether MMI enhances collateral circulation formation through this secreted factor. We cocultured THP-1-derived macrophages with human umbilical vein endothelial cells (HUVECs) to model angiogenesis *in vitro* (Figure 4F). Compared with DMSO-treated control macrophages, MMI-treated macrophages significantly increased angiogenesis, as evidenced by an increased number of branching points and total length of the endothelial cell network (Figure 4G-H). Importantly, VEGF secretion from cocultured macrophages was markedly increased in the MMI-treated groups, as confirmed by ELISA (Figure 4I).* In vivo*, MMI treatment resulted in prominent EC proliferation in the watershed area of the heart of MI mice on Day 3 after MI, as observed using immunofluorescence staining for Ki67^+^CD31^+^ cells (Figure 4J-K). These results suggest that MMI promotes angiogenesis and CC formation through M2 macrophage polarization and VEGF secretion.

### MMI facilitates M2-like macrophage polarization via ferroptosis

**Figure 5:**
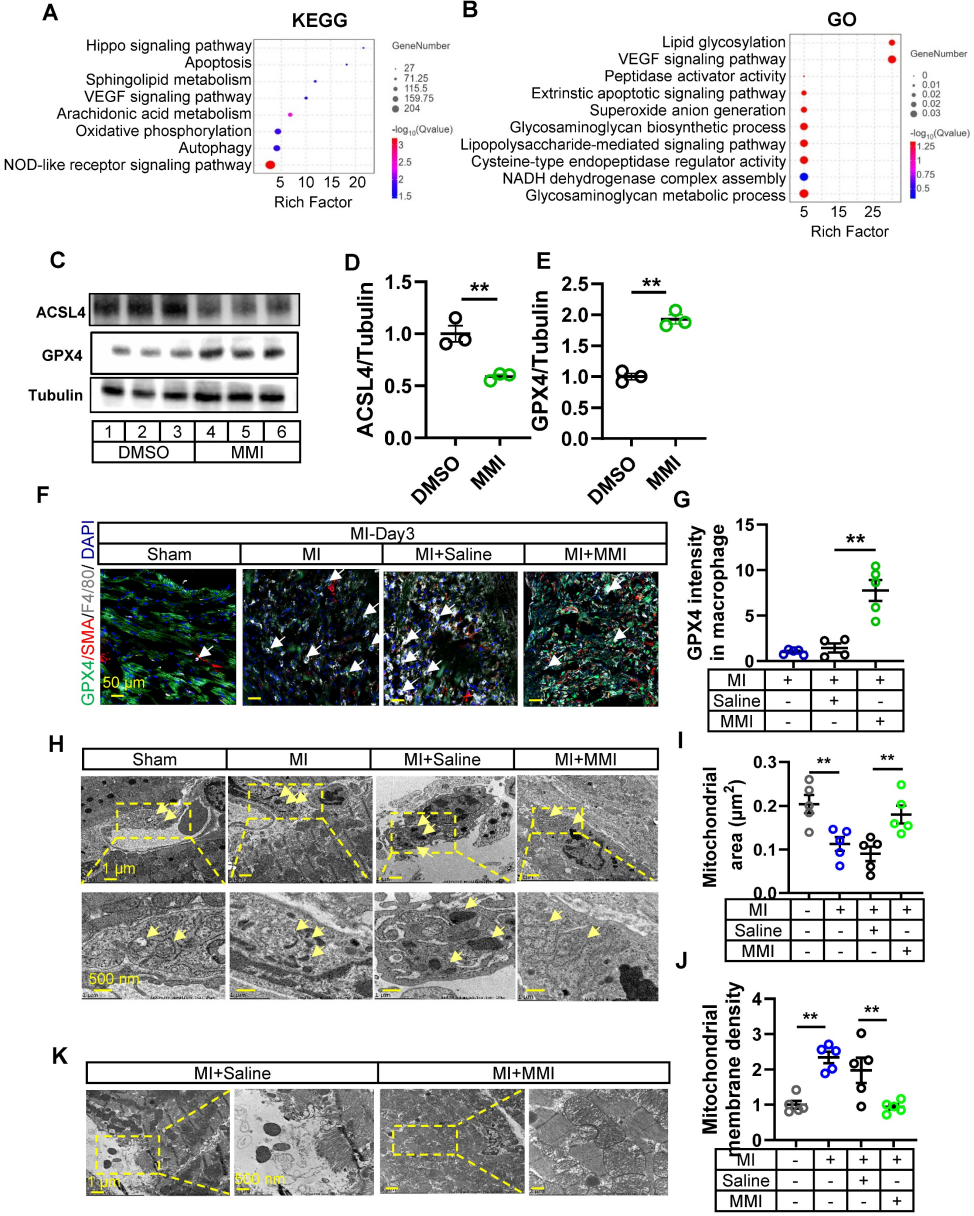
** MMI facilitates M2-like macrophage polarization via ferroptosis. A** and** B.** KEGG **(A)** and GO **(B)** analysis of DEGs between macrophages sorted from MMI and saline treated heart after MI injury by transcriptional RNA sequence. n = 3 mice for each group. **C-E**. Representative western blot images **(C)** and quantification **(D and E)** of GPx4, ACSL4 and tubulin in THP-1 derived M1 macrophage with DMSO or 5 μM MMI (lane 1, 2 and 3, DMSO; lane 4, 5 and 6, MMI), n = 3 independent experiment. **F** and **G**. Representative confocal images (F) and Quantification (G) of GPX4 (green), SMA (red) and F4/80 (white) immunostaining in watershed area of heart with indicated treatment from at 3 days after MI injury (the white arrow pointed to the GPX4^+^ macrophage cells, scale bar, 50 μm). **H-J**. Representative transmission electron microscope (TEM) images of macrophage (H) and quantification of mitochondrial area (μm^2^) (I) and membrane density (J) at 3 days after MI. Yellow area depicted mitochondria area with 10* (left) and 20* (right) in macrophage of watershed area collected from mice MI heart with saline or MMI treatment, yellow arrow pointed to the mitochondria in macrophage, scale bar, 1 μm (up) and 500 nm (down). n = 5 mice for each group. **K.** Representative TEM image of cardiomyocyte in watershed of heart with MI injury following saline and MMI treatment, scale bar, 1 μm (left) and 500 nm (right). One-way ANOVA with Tukey multiple comparisons test in (G, I and J). Student's *t* test (two-tailed, unpaired) in (D, E) (**p<0.01). Each dot represents a single mouse. Data are represented as mean ± SEM.

We investigated the mechanism by which MMI facilitates M2-like macrophage polarization by isolating macrophages from the hearts of control or MMI-treated mice 3 days post-MI and performed bulk RNA sequencing. The KEGG pathway analysis and GO analysis revealed several ferroptosis-related pathways, including autophagy, peptidase activator activity and NADH dehydrogenase complex assembly (Figure 5A-B). These findings prompted us to explore the effect of MMI on macrophage ferroptosis. *In vitro*, MMI treatment of THP-1-derived macrophages reduced the levels of ACSL4, a proferroptosis marker, and simultaneously increased the expression of GPX4, a key antiferroptosis marker (Figure 5C-E) 28, 29.

We assessed the GPX4 intensity in F4/80^+^ macrophages in the MI model to further examine the role of MMI in ferroptosis *in vivo*. As expected, GPX4 levels were reduced in control MI hearts, but MMI treatment restored GPX4 expression in macrophages (Figure 5F-G). Additionally, a transmission electron microscopy (TEM) analysis of heart tissue from MI mice revealed distinctive mitochondrial alterations, including an increased mitochondrial volume and increased membrane density, in the control group. In contrast, MMI-treated hearts presented an increased mitochondrial volume and lower membrane density (Figure 5H-J). These results suggest that MMI protects macrophages from oxidative stress-induced damage via ferroptosis modulation. Furthermore, a cardiomyocyte ultrastructure analysis showed a disruption of Z-lines, loss of myonuclear structure, and mitochondrial swelling in control MI hearts. However, MMI-treated hearts exhibited a normal myonuclear arrangement and preserved mitochondrial morphology (Figure 5K), suggesting that MMI confers protective effects on oxidative stress-induced damage in the myocardium.

### The MMI-induced ferroptosis reduction depends on the MAPK1/ reactive oxygen species (ROS) axis

**Figure 6:**
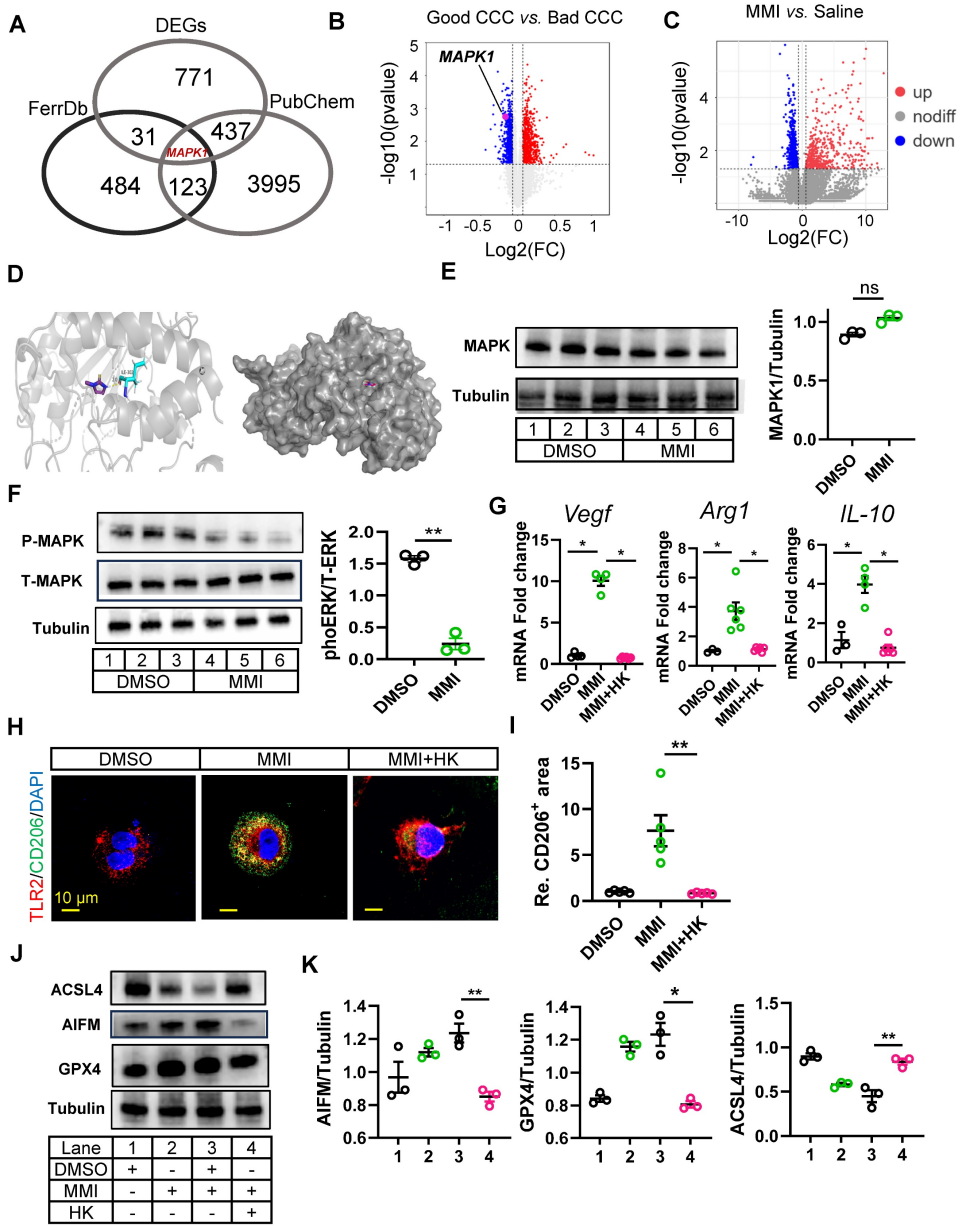
** MAPK1 mediated MMI-induced M2-like polarization by ERK phosphorylation. A.** Venn diagrams of shared targets among ferroptosis data (FerrDb). PubChem and DEGs between macrophages sorted from heart with saline and MMI. **B.** MAPK1 indicated as pink dot in the volcano plot of DEGs from the public dataset GSE7547. **C.** MAPK1 is indicated as red dot in the volcano plot of DEGs from the bulk RNA-seq of cardiac tissue with MI injury treated saline and MMI. **D.** The predicted docking pose of MMI with MAPK1 domain. The protein residues bind to MMI is colored in blue, hydrogen bonds are shown in yellow dashed lines and the number represent the length of hydrogen bond connecting two atoms. **E.** Western blot analysis of MAPK1 (left) and quantification of protein levels (right) in THP-1 derived macrophage treated with DMSO or MAPK1, n = 3 independent experiment. **F.** Western blot image and quantification of phos-MAPK1 and total-MAPK1 in THP-1 derived macrophage treated with DMSO or MAPK1, n = 3 independent experiment. **G.** Quantitative RT-PCR analysis of Arg-1, IL-10 and VEGF in macrophage administrated with MMI or MMI and HK combination. **H.** Representative confocal images of TLR2 (red) and CD206 (green) immunofluorescent staining in THP-1 derived macrophage with indicated treatment (scale bar, 10 μm). **I**. Quantification of CD206 area from **(H). J** and **K**. Representative western blot images **(J)** and quantification **(K)** of GPx4, AIFM, ACSL4 and tubulin in THP-1 derived M1 macrophage with indicated treatment, n = 3 independent experiment. One-way ANOVA with Tukey multiple comparisons test in **(G)**, **(I)** and **(K)** (*p<0.05, **p<0.01). Student's *t* test (two-tailed, unpaired) in **(E)** and **(F)** (ns *p* > 0.05, **p<0.01). Data are represented as mean ± SEM.

Previous studies have highlighted the role of ferroptosis in regulating macrophage polarization, and our findings suggest that MMI-mediated M2-like macrophage polarization is influenced by ferroptosis. Specifically, the application of a ferroptosis activator significantly attenuated the MMI-induced expression of M2-like markers (Figure S4A), suggesting that MMI modulates macrophage polarization through the ferroptosis pathway. We further explored the molecular mechanisms underlying the effect of MMI on CCC formation by performing an overlay analysis using the FerrDb database (http://www.zhounan.org/ferrdb), which associated ferroptosis targets with DEGs from bulk RNA sequencing data between saline- and MMI-treated hearts. This analysis revealed MAPK1 as a potential target (Figure 6A). Notably, MAPK1 expression was downregulated in the good CCC cohort (GSE7547), although it was not significantly altered in the MMI versus saline comparison in our RNA-seq data (Figure 6B-C). Molecular docking studies confirmed a direct interaction between MMI and MAPK1 (binding energy = -4.53 kcal/mol, with one hydrogen bond) (Figure 6D).

*In vitro*, the treatment of THP-1-derived macrophages with 5 μM MMI did not affect MAPK1 protein levels (Figure 6E). We investigated whether MMI affects MAPK1 phosphorylation and observed that MMI reduced phosphorylated MAPK1 levels (Figure 6F).

Next, we explored whether the activation of MAPK1 interferes with MMI-induced M2-like polarization. Compared with MMI treatment alone, cotreatment with the MAPK1 activator honokiol (HK) significantly decreased the expression of M2-like markers (*Vegf, Arg1* and IL-10) (Figure 6G). Additionally, the proportion of TLR2^+^CD206^-^ macrophages was significantly increased, whereas the proportion of TLR2^-^CD206^+^ macrophages was markedly decreased in the presence of HK and MMI (Figure 6H-I). Furthermore, compared with MMI alone, HK combined with MMI impaired the angiogenic potential of macrophages *in vitro*, as evidenced by decreased HUVEC angiogenesis and VEGF secretion (Figure S4B-E). Finally, we assessed the levels of ferroptosis-related markers and found that the combination of MMI and HK significantly induced ACSL4 expression and inhibited AIFM and GPX4 protein expression, indicating that HK could counteract the inhibitory effect of MMI on ferroptosis (Figure 6J-K). These findings collectively indicate that MMI-mediated M2-like macrophage polarization depends on MAPK1 signaling and its modulation of ferroptosis.

Previous studies have reported that inhibition of MAPK1 phosphorylation can reduce ROS levels 30, which in turn may decrease ferroptosis 31. To elucidate whether the inhibition of MAPK1 phosphorylation mediated by MMI prevents ferroptosis by suppressing ROS, we measured ROS level and malondialdehyde (MDA) levels (a classical marker of lipid peroxidation) in M1 macrophages with DMSO, MMI, and MMI combined with HK. We found that ROS activity (Figure S5A) and MDA level (Figure S5B) in M1 macrophages was significantly reduced in MMI treatment, but HK could restore the ROS level. In the murine MI model, flow cytometric analysis demonstrated a significant increase in ROS levels in cardiac macrophages following ischemic injury. MMI treatment attenuated these elevated ROS levels, whereas the administration of HK counteracted this effect (Figure S5C-E). Furthermore, we treated M1 macrophages with both MMI and H₂O₂ to determine whether ROS activity could block MMI/MAPK1 mediated ferroptosis inhibition. Compared with MMI treatment alone, cotreatment with H₂O₂ led to significant upregulation of ferroptosis-promoting proteins such as ALOX15 and ACSL4 and marked downregulation of ferroptosis-inhibiting proteins such as GPX4 and AIFM. Moreover, a MAPK1 inhibitor similarly suppressed ACSL4 expression and enhanced GPX4 expression—an effect that was likewise reversed by H₂O₂ treatment. Additionally, there was no significant difference in the expression of ferroptosis-related proteins between the MMI with HK and MMI/HK/H₂O₂ groups (Figure S5F-I). These findings suggest that MMI inhibits ferroptosis primarily through suppression of the MAPK1/ROS axis.

### Honokiol reverses the promotion of collateral circulation in the MI mouse heart mediated by MMI

**Figure 7:**
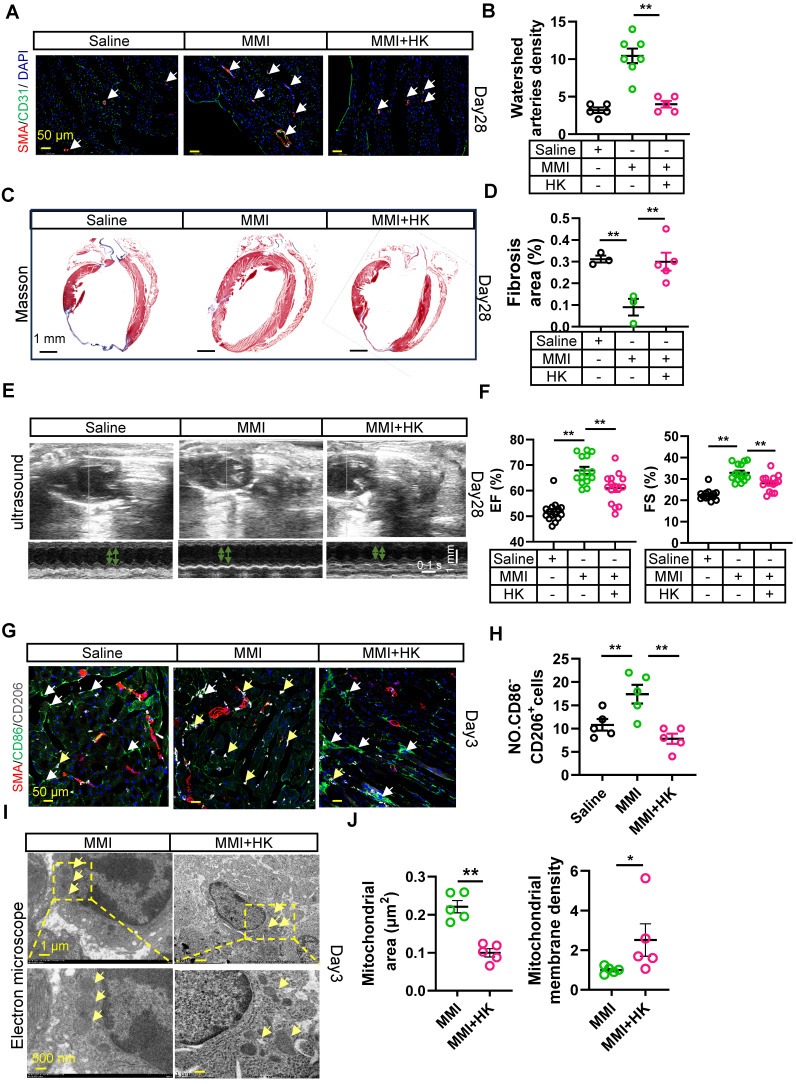
** Honokiol reverse MMI's promotion effects on CCC in MI mouse heart. A** and **B**. Representative images of SMA (red) and CD31 (green) immunofluorescent staining **(A)** and quantification of watershed arteries density **(B)** at 28 days after MI (white arrow indicated the collateral arteries in watershed, scale bar, 50 μm). **C** and **D**. Representative images of Masson staining **(C)** and Quantification of fibrosis area (%) **(D)** at 28 days after MI (fibrosis (blue); scale bar, 1 mm). **E.** Representative images of UCG to evaluate cardiac function at 28 days after MI (scale bar 1 mm, 0.1 s). **F.** Quantification of EF (%) and FS (%) from E. **G.** Representative confocal images of SMA (red), CD86 (green) and CD206 (white) immunofluorescent staining in watershed area at day 3 after MI (the white arrow points to CD86^+^CD206^-^ cells and yellow arrow point to CD86^-^CD206^+^ cells, scale bar, 50 μm). **H**. The quantification of No. CD86^-^CD206^+^ cells (M2-like macrophage) from **(G)**. **I** and** J.** Representative images of TEM **(I)** and Quantification **(J)** of mitochondrial area and membrane density at 3 days after MI (scale bar, 1 μm (up), 500 nm(down)). One-way ANOVA with Tukey multiple comparisons test in **(B)**, **(D)**, **(F)**, **(H)** (**p<0.01). Student's *t* test (two-tailed, unpaired) in **(J)** (*p<0.05, **p<0.01). Each dot represents a single mouse. Data are represented as mean ± SEM.

We next investigated whether HK could modulate collateral circulation formation in a murine MI model. *In vivo* rescue experiments were conducted, where MMI and HK (10 mg/kg) were administered intraperitoneally daily for one week after MI. Honokiol effectively mitigated the MMI-induced CC formation, as evidenced by immunofluorescence staining for α-SMA/CD31, which revealed a reduction in both the CC diameter and density at 28 days following MI (Figure 7A-B). Additionally, Masson's trichrome staining revealed that honokiol significantly increased the fibrotic scar area in MMI + HK-treated mice compared with the hearts of MI mice treated with saline (Figure 7C-D). UCG assessments of cardiac function at 28 days post-MI further corroborated these findings, showing that EF (%) was significantly altered in mice treated with MMI, with or without honokiol, compared with saline-treated controls (Figure 7E-F). On day 3 post-MI, the proportion of M2-like macrophages (CD86^+^CD206^-^) was significantly greater in MMI + HK-treated mice than in those treated with MMI alone (Figure 7G-H). Moreover, mitochondrial morphology was altered in the MI hearts of these mice: while MMI treatment alone resulted in an increased mitochondrial volume and membrane density, cotreatment with MMI and HK led to a reduction in the mitochondrial volume and an increase in the membrane density (Figure 7I-J). These results suggest that honokiol reverses the effects of MMI on CC formation and mitochondrial morphology in the context of MI.

## Discussion

In this study, we investigated the therapeutic potential of MMI in promoting CCC formation in a mouse MI model, focusing on its effects on macrophage polarization and ferroptosis. Our findings suggest that MMI facilitates M2-like macrophage polarization, which is crucial for CCC formation, by modulating ferroptosis through the MAPK1/ROS axis. This study highlights the potential of targeting ferroptosis and macrophage polarization as therapeutic strategies to enhance postinfarction cardiac repair and vascular regeneration.

Current consensus posits that CCC evolves through two distinct phases following MI: initial recruitment of preexisting anastomotic vessels and subsequent maturation into functional conduits via progressive remodeling 32. By employing the below phase-specific methodologies, we demonstrate MMI's capacity to enhance collateral-dependent myocardial salvage throughout post-infarct recovery. Our findings identify MMI as a dual-phase therapeutic agent that confers early cardioprotection via a reduced infarct area (TTC), At the advanced stage of MI, it can coordinately alleviate fibrosis (detected by Masson trichrome staining) and promote functional recovery (EF%). Macrophage polarization plays a pivotal role in tissue repair following MI 33. Previous studies have demonstrated that M2-like macrophages promote tissue regeneration and angiogenesis through the secretion of proangiogenic factors such as VEGF 34. In our study, MMI treatment increased the polarization of macrophages from the M1-like phenotype to the M2-like phenotype, as evidenced by changes in the expression of characteristic markers such as IL-6, IL-1β (M1-like) and VEGF (M2-like) 35. This M2 polarization is particularly important in the context of MI, where macrophages contribute to both wound healing and the formation of collateral blood vessels that compensate for the loss of myocardial perfusion 36, 37.

The molecular mechanisms underlying MMI-induced macrophage polarization were further explored through bulk RNA sequencing and functional pathway analyses, revealing that ferroptosis-related pathways play crucial roles in mediating this process. Ferroptosis, a form of regulated cell death driven by iron-dependent lipid peroxidation, has been implicated in various pathological conditions, including ischemia‒reperfusion injury and neurodegenerative diseases 38-40. In our study, ferroptosis was identified as the key mechanism through which MMI promotes macrophage polarization toward the M2 phenotype, a process vital for CCC formation. Specifically, MMI reduced the levels of the proferroptosis marker ACSL4 while increasing the expression of the antiferroptosis proteins AIFM and GPX4, thus protecting macrophages from ferroptotic cell death 28, 29. This inhibition of ferroptosis facilitated macrophage survival and induced a shift toward the M2 phenotype, which is known to promote endothelial cell proliferation and migration and the formation of collateral vessels. The ability of MMI to regulate ferroptosis and macrophage polarization highlights a novel therapeutic pathway for enhancing CCC formation in individuals with CHD.

Furthermore, our study identified MAPK1 as a critical mediator of MMI-induced M2-like polarization. Using a combination of gene expression profiling and molecular docking, we showed that MMI directly interacts with MAPK1, leading to decreased MAPK1 phosphorylation and activation and enhanced M2-like macrophage polarization. Moreover, we observed that the inhibition of MAPK1 phosphorylation suppresses ferroptosis by blocking the ROS through H₂O₂ intervention experiments. Our findings suggest that inhibition of the MAPK1/ROS axis may be a novel mechanism by which MMI promotes CC formation following MI 41.

The therapeutic implications of these findings are significant, as enhancing macrophage polarization toward an M2-like phenotype may represent a strategy to improve vascular repair after MI. Moreover, we showed that honokiol, an activator of MAPK1, reversed the effects of MMI on CC formation, further supporting the idea that MAPK1 plays a central role in this process 42, 43. These findings highlight the potential of combining MMI with other therapeutic agents to fine-tune macrophage polarization and improve myocardial recovery.

While our findings establish MMI as a promising modulator of ferroptosis-dependent macrophage polarization for post-MI collateral remodeling, two aspects warrant further translational exploration. First, although our study focused on the localized cardioprotective mechanisms of MMI at a low therapeutic dose (monitored for hepatic/renal safety), its systemic antithyroid effects and optimal dosing windows require validation in species with thyroid physiology analogous to that of humans. Second, while the preclinical toxicity screen revealed no acute organ dysfunction of the administered regimen, comprehensive dose-escalation studies and large-animal models will be essential to refine its therapeutic index before clinical consideration. These targeted investigations provide mechanistic insights into the cardiac-specific benefits of MMI with its established pharmacological profile, accelerating its potential repurposing for ischemic cardiomyopathy.

In summary, our study demonstrated that MMI promotes CCC in an MI mouse model by modulating macrophage polarization and ferroptosis through the MAPK1/ROS axis. These findings provide a foundation for future investigations into the use of MMI as a therapeutic agent for improving vascular regeneration and cardiac repair in individuals with ischemic heart disease.

## Supplementary Material

Supplementary materials and methods, figures and tables.

## Abbreviations

CHD: coronary heart disease
MI: myocardial infarction
CCC: coronary collateral circulation
CC: collateral circulation
ECs: endothelial cells
CMAP: connectivity map
MMI: methimazole
DEGs: differentially expressed genes
PBMCs: peripheral blood mononuclear cells
CFI: collateral flow index
LVEF: left ventricular ejection fractions
LAD: left anterior descending
UCG: ultrasound cardiography
FS: fractional shortening
EF: ejection fraction
SMILES: simplified molecular input line entry system
KEGG: Kyoto Encyclopedia of Genes and Genomes
GO: Gene Ontology
ROS: reactive oxygen species
HUVECs: human umbilical vein endothelial cells
DMSO: dimethyl sulfoxide
HK: Honokiol
CAD: coronary artery disease
