# Supplementary material for: Repurposing methimazole to promote coronary collateral circulation through MAPK1-mediated macrophage polarization via ferroptosis
Source: Theranostics. 2025 Jun 9;15(14):6686–701. doi: 10.7150/thno.111606 (PMC12203669; doi:10.7150/thno.111606)
Supplement: Supplementary file 1 — Supplementary materials and methods, figures and tables. [file thnov15p6686s1.pdf]

Supplementary materials

1. **Materials and methods**
2. **Supplemental Tables**
3. **Supplemental Figures**

1. Materials and methods

Drug screening and molecular docking

The expression data from the monocytes of individuals were retrieved from the GEO database (https://www.ncbi.nlm.nih.gov/geo/) (GSE7547 and GSE11947) and analyzed via the GEO2R platform to identify differentially expressed genes (DEGs). Genes with p values < 0.05 and log2FC > 0.1 were classified as upregulated DEGs, whereas those with p values < 0.05 and log2FC < -0.1 were categorized as downregulated DEGs. The shared genes from these two datasets were uploaded to CMap (https://clue.io/) with the query parameters "GENE expression (L1000)" and "dataset 1.0" to identify potential therapeutics that promote CCC. The results included the compound name, connectivity score, replicate correlation coefficient (CC), transcriptional activity score (TAS) and signature strength (SS). Compounds with a negative connectivity score, CC > 0.2, TAS > 0.15 and SS > 100 were considered promising candidates. The drug list is presented in Table S2. Methimazole (MMI) was subsequently identified as the most promising candidate for promoting coronary collateral circulation (CCC). Potential drug targets for MMI were identified by examining chemical–gene co-occurrences in the literature, analyzing protein-bound 3D structures, and exploring BioAssay data in PubChem (https://pubchem.ncbi.nlm.nih.gov/). MAPK1 was identified as the primary target of MMI. Molecular docking between selumetinib and MAPK1 was conducted using Autodock4 (https://autodock.scripps.edu). The 3D conformer of the MMI was retrieved from PubChem (https://pubchem.ncbi.nlm.nih.gov/compound/1349907). The MAPK1 domain was obtained from X-ray crystal structure 5v62 available in the Protein Data Bank (PDB, https://www.rcsb.org/structure/5v62). The docking procedure followed the AutoDock protocol, and the default value was adopted.

Study population

This retrospective cohort study aimed to evaluate the impact of MMI on heart function in patients with severe coronary artery disease (CAD). A total of 32 patients with CAD were included, 23 of whom were taking MMI and 9 of whom were not. The inclusion criterion was patients with significant coronary artery stenosis (≥ 70% in three major vessels). The exclusion criteria included patients with a malignancy, diabetes mellitus, and acute infections. Patients' data, including demographic information, medication history, inflammatory cell counts,

C-reactive protein level and clinical outcomes, were retrospectively collected from electronic medical records. **Clinical baseline information of these patients was shown in table S1.** The endpoint was the left ventricular ejection fraction (LVEF). Statistical comparisons between the two groups were performed using *t* tests and chi-square tests, with adjustment for potential confounders through logistic regression models.

Ethics statement

The animal care and experimental protocols were approved by the Ethics Committee of Xiangya Hospital, Central South University, and adhered to the National Institutes of Health (NIH) Guidelines for the Care and Use of Laboratory Animals.

Animal myocardial infarction (MI) model

Male C57BL/6 mice (8–10 weeks old) were obtained from Central South University, Changsha, Hunan Province, China. The mice were housed in a sterile environment with free access to water and standard rodent chow and maintained on a 12:12 h light–dark cycle. Isoflurane (2-3% in 100% oxygen, Shenzhen Reward Life Technology Co., Ltd., China, R510-22-8) was administered as an inhalation anesthetic. Anesthesia was confirmed by the absence of the pedal reflex and was continuously monitored through the respiratory rate, depth, and lack of response to noxious stimuli. The mice were maintained under anesthesia until the procedure was completed. Following anesthesia induction and intubation for mechanical ventilation, MI was induced by ligating the left anterior descending coronary artery with a 10–0 nylon suture [1, 2]. An ultrasound cardiogram (UCG) was conducted at 28 days post-MI using a Mindray ZONARE ultrasound machine equipped with a high-frequency probe for small animals (frequency: 70 Hz). The mice were lightly anesthetized with isoflurane**,** and their chest fur was shaved to optimize probe contact. Echocardiographic images were acquired from multiple views, including the parasternal long-axis and short-axis perspectives, to assess cardiac structure and function. Key parameters, including fractional shortening% (FS%) and ejection fraction% (EF%), were measured to evaluate myocardial function following infarction.

Following all the experiments and procedures, euthanasia was induced using carbon dioxide (CO_2) in a gradual-fill manner. CO_2 was administered at a controlled rate of 20–30% of the chamber volume per minute to minimize distress. After unconsciousness was confirmed, CO_2 flow was maintained for an additional 1–2 minutes to ensure complete euthanasia.

Drug administration *in vivo*

The mice were randomly assigned to five groups: the sham operation (sham), MI, MI + saline, MI + MMI, and MI + MMI + honokiol (HK) groups. MMI was administered at a dose of 5 mg/kg on Days 0, 1, 2, 3, 7, 10, and 14, whereas

HK was administered at a dose of 10 mg/kg per day for one week. The MI + saline group received physiological saline injections at the same frequency.

Cell culture and treatment

Human umbilical vein endothelial cells (HUVECs) and human monocytes (THP-1) were used for *in vitro* investigations. HUVECs (ScienCell Cat #8000) were cultured in endothelial cell medium (ECM; ScienCell Cat #1001) supplemented with 10% FBS, 1% penicillin–streptomycin, and 1% growth factors (ScienCell Cat #1052). THP-1 cells (SCSP-567) were cultured in RPMI 1640 medium supplemented with 10% FBS, 0.05 mM β-mercaptoethanol and 1% penicillin–streptomycin. HUVECs at passages 3 to 6 were utilized in this study. The cells were maintained at 37 °C in a humidified incubator with 5% CO_2. The culture medium was replaced every other day. The cells were subcultured at a 1:3 ratio upon reaching 80% confluence. THP-1 cells were seeded into 6-well plates at a density of 5×10^5 cells/mL. Upon reaching approximately 80% confluence, the cells were treated with 100 nmol/L PMA for 48 hours in complete culture medium to induce their differentiation into macrophages. The cells were subsequently cultured for an additional 24 hours in PMA-free medium to achieve M0 polarization. Polarization induction was deemed complete once more than 80% of the suspended cells adhered to the plate.

Microcomputed tomography (micro-CT) angiography

Microfil perfusion of mouse heart coronary arteries was conducted as previously described [3]. The mice were anesthetized with 2–3% isoflurane, followed by an intravenous injection of 50 μL of 1% heparin. After euthanasia via CO_2 exposure, the thoracic aortas of the mice were perfused with PBS containing sodium nitroprusside and adenosine, followed by paraformaldehyde (PFA) perfusion for tissue fixation. The coronary vessels were infused with Microfil (MV-122, Flowtech, Inc.) and analyzed using a Hiscan XM Micro CT system (Suzhou Hiscan Information Technology Co., Ltd.). The X-ray tube was operated at 60 kV and 134 μA, with images acquired at an 8 μm resolution. Image reconstruction was conducted using Hiscan Reconstruct software (Version 3.0; Suzhou Hiscan Information Technology Co., Ltd.), and the analysis was performed using Hiscan Analyzer software (Version 3.0; Suzhou Hiscan Information Technology Co., Ltd.). The watershed region between the left anterior descending artery and the right coronary artery was locally magnified to assess revascularization following MI. The diameters of watershed arteries and arterial density were quantified to evaluate revascularization.

Western blot analysis

Western blot analysis was conducted according to established protocols. Briefly, cells or heart tissues were lysed in buffer containing 2% SDS, 10% glycerol, and Tris-HCl (pH 6.8) through sonication. The total protein concentration was quantified using a BCA assay kit, and the samples were mixed with SDS–PAGE

sample loading buffer (Beyotime, China) for analysis. Proteins were resolved via SDS–polyacrylamide gel electrophoresis (SDS–PAGE) and transferred to a 0.45 μm PVDF membrane for immunoblotting. The primary antibodies used included anti-TNF-α, anti-IL-1β, anti-IL-6, anti-IL-10, anti-VEGF, anti-p-ERK1/2, and anti-T-ERK1/2 (1:1000), along with anti-GAPDH and anti-β-Tubulin antibodies diluted 1:2000. The secondary antibodies used were rabbit anti-mouse IgG HRP and goat anti-rabbit IgG HRP, both at a 1:5000 dilution (Abcam, UK). The results are representative of at least three independent experiments. The integrated optical density (IOD) of each band was quantified using ImageJ software, and relative protein expression was calculated as the ratio to β-tubulin or GAPDH IOD.

Real-time quantitative PCR (RT–qPCR)

For RT–qPCR, cDNA synthesis was conducted using the PrimeScript RT reagent kit (TaKaRa, Japan) according to the manufacturer's protocol, which involved 1 μg of RNA, reverse transcriptase, oligo(dT) primers, and reaction buffer. The synthesized cDNA was diluted to an appropriate working concentration for subsequent qPCR analysis. Specific primers for the target genes were designed using the Primer Bank (https://pga.mgh.harvard.edu/primerbank/) and are listed in Table S4. These primers were optimized for melting temperature and verified to be free of secondary structures. qPCR was conducted using SYBR Green PCR Master Mix (Applied Biosystems) in a total reaction volume of 20 μL, consisting of 10 μL of SYBR Green mix, 1 μL of each primer (10 μM), 2 μL of diluted cDNA, and 6 μL of RNase-free water. qPCR was performed on a real-time PCR system, starting with initial denaturation at 95 °C for 10 minutes, followed by 40 cycles of 95 °C for 15 seconds and 60 °C for 1 minute. A melting curve analysis was performed after amplification to confirm the specificity of the amplified products. Relative gene expression was quantified using the $2^{-\Delta\Delta Ct}$ method, with normalization to the expression of housekeeping genes.

Coculture of HUVECs and macrophages and *in vitro* angiogenesis

THP-1 cells and HUVECs were cocultured to study angiogenesis *in vitro*, as previously described [4]. HUVECs (1×10^5 cells) were seeded into the lower wells of a 6-well Transwell plate. THP-1 cells at the same density were seeded into the upper chamber of a Transwell plate and maintained separately in another 6-well plate. Following stimulation with PMA for 48 hours, the THP-1 cells were cultured for an additional 24 hours in PMA-free medium to allow complete differentiation into a single layer of macrophages. The upper chamber containing macrophages was reinserted into the Transwell plate, where HUVECs were cultured in the bottom wells. After coculture, a tube formation assay was performed with HUVECs to evaluate angiogenesis. Capillary-like structures (lumens) were assessed using HUVECs cultured in 96-well plates precoated with Matrigel. HUVECs (3×10^4 cells/well) were seeded onto Matrigel

(50 µL/well) in endothelial cell medium (ECM). The organization of HUVECs on Matrigel was recorded after 24 hours of incubation at 37 °C using a phase-contrast microscope (Leica DFC7000T) at 40× magnification. The total tube length was quantified by calculating the mean pixel density in images of microscopic fields using ImageJ software.

PGSK probe assay

The aim of this study was to use PGSK probes to assess the inhibitory effect of MMI on ferroptosis in macrophages. PGSK probes were then added to the cultures at a final concentration of 10 μM and incubated for an additional 30 minutes to allow for probe uptake. The levels of ferroptosis-related markers, including lipid peroxidation and iron accumulation, were detected using fluorescence microscopy and flow cytometry. The fluorescence intensity of PGSK staining was quantified to assess ferroptosis levels. The statistical analysis was performed using one-way ANOVA with Tukey's post hoc test, with a significance threshold of $*p < 0.05$.

Enzyme-linked immunosorbent assay (ELISA)

VEGF levels were measured using a mouse VEGF ELISA kit (Sino Biological, China), according to the manufacturer's instructions. Briefly, standards and samples were loaded into the wells, and detection antibodies were subsequently added. The plate was incubated and washed to remove unbound substances, and a substrate solution was applied to initiate color development. After adequate color development, a stop solution was applied to terminate the reaction. The optical density (OD) at 450 nm was promptly measured using a microplate reader (ELx800, Bio-Tek). Measurements were performed in duplicate, and data were analyzed using a standard curve generated from known VEGF concentrations.

Pathological examination

Masson staining

The mice were anesthetized with 2–3% isoflurane, followed by cervical dislocation for euthanasia. Perfusion was performed through the thoracic aorta using PBS containing sodium nitroprusside and adenosine, followed by fixation with PFA, after which the heart was carefully excised for tissue collection. Hearts were fixed with 4% paraformaldehyde for 48 hours and subsequently embedded in paraffin. Paraffin sections were prepared at a thickness of 4 μm using a Leica microtome (Leica RM2125 RTS). Paraffin-embedded heart sections were deparaffinized and stained with a Masson’s trichrome staining kit (Servicbio, China). The sections were incubated in Weigert's iron hematoxylin for 5 minutes and then in acid fuchsin for 5–10 minutes. Phosphomolybdic acid was applied for 3–5 minutes, followed by staining with aniline blue for 5 minutes. The sections were treated with 1% acetic acid, dehydrated in ethanol, cleared in xylene, and mounted with neutral resin. Necrotic areas were captured at 20×

magnification using a microscope (Leica DFC7000 T). Fibrosis (blue staining) was quantified using ImageJ software (NIH, Germany), with at least 8 images per heart analyzed in a double-blind manner.

Hematoxylin and eosin (H&E) staining

The mice were anesthetized with 2–3% isoflurane, followed by cervical dislocation for euthanasia. Perfusion through the thoracic aorta was conducted with PBS containing sodium nitroprusside and adenosine, followed by fixation with PFA. The kidney and liver were then carefully excised for tissue collection. The kidney and liver were fixed with 4% paraformaldehyde for 48 hours and embedded in paraffin. Paraffin sections were prepared at a thickness of 4 μm using a Leica microtome (Leica RM2125 RTS). Paraffin-embedded kidney and liver sections were deparaffinized and stained according to a H&E staining protocol. The sections were incubated with hematoxylin for 5–10 minutes, followed by rinsing under running water for 5 minutes to remove excess dye. The sections were subsequently stained with eosin for 1–3 minutes.

The sections were then dehydrated in graded ethanol solutions, cleared in xylene, and mounted with neutral resin. Necrotic areas were quantified at 40× magnification using a microscope (Leica DFC7000 T), with measurements analyzed using ImageJ software (NIH, Germany). At least 8 images per tissue sample were evaluated in a double-blind manner.

Immunostaining of heart cross-sections

The mice were anesthetized with 2–3% isoflurane, followed by cervical dislocation for euthanasia. Perfusion through the thoracic aorta was performed with PBS containing sodium nitroprusside and adenosine, followed by tissue fixation with PFA. The hearts were then carefully excised for collection. Hearts were fixed with 4% paraformaldehyde for 24 hours and embedded in optimal cutting temperature (OCT) compound. Frozen sections were prepared at a thickness of 10 μm using a Leica microtome (HistoCore BIOCUT). The frozen heart sections were washed with PBS to remove the OCT, boiled in EDTA–citrate buffer (pH 8.0) for 2 minutes, and then cooled to room temperature. The sections were permeabilized with 0.3% Triton X-100 in PBS for 30 minutes and subsequently blocked with 10% donkey serum and 3% BSA in PBS for 1 hour. The sections were incubated with primary antibodies overnight at 4 °C. The primary antibody dilutions used were as follows: anti-CD31 (1:100), anti-SMA (1:200), anti-CD68 (1:100), anti-CD206 (1:200), anti-CD86 (1:100), anti-F4/80 (1:100), anti-MAPK1 (1:100), and anti-Ki67 (1:100). Following washes to remove the unbound primary antibody, the sections were incubated with the appropriate secondary antibodies for 1 hour. After an incubation with secondary antibodies and subsequent washing, the sections were mounted with DAPI-containing mounting medium. Images were acquired with a Zeiss LSM 900 confocal microscope (Zeiss, Germany) at magnifications of 10× or 40×, and statistical analyses were conducted using Zen blue software.

Transmission electron microscopy (TEM)

The animals were anesthetized and transcardially perfused with saline, followed by 4% paraformaldehyde after surgery. Heart tissues from the injury center were immediately collected and fixed with 2.5% glutaraldehyde for 4 hours. The samples (1 × 1 × 3 mm^3) were sliced, double fixed with 2.5% glutaraldehyde, and shipped overnight at ambient temperature to the TEM laboratory (Wuhan Servicebio Technology Co., Ltd.) for further processing. During preparation, the samples were washed with Millonig's phosphate buffer (pH 7.4), incubated in 1% osmic acid (Ted Pella Inc., #18456) for 2 hours, and washed again. The samples were dehydrated at room temperature in an acetone gradient (30%, 50%, 70%, 80%, 95% and 100%, each for 20 minutes, followed by two 15-minute incubations with 100% acetone). The samples were soaked in a 1:1 mixture of acetone and 812 embedding agent for 2–4 hours and embedded in a 1:2 mixture of acetone and 812 embedding agent overnight at 37 °C. Pure 812 embedding medium was placed in embedding molds, and the samples were inserted. The molds were then placed in a 37 °C oven and incubated for 5–8 hours. After embedding, the samples were transferred to a 60 °C oven for polymerization for 48 hours, and the resin blocks were retrieved for further use. The resin blocks were sectioned into semithin slices (1.5 μm) using a semithin sectioning machine (Leica UC7) and stained with toluidine blue for light microscopy localization. Ultrathin sections (60–80 nm) were subsequently prepared using an ultrathin sectioning machine, and the sections were subsequently transferred onto 150-mesh copper grids. The copper grids were stained in a dark room with 2% uranyl acetate in an alcohol solution for 8 minutes, followed by three washes with 70% ethanol and three washes with ultrapure water. The sections were then stained with a 2.6% lead citrate solution in a CO_2-free environment for 8 minutes, followed by three washes with ultrapure water. The grids were gently blotted with filter paper and left to dry overnight at room temperature. Observations were made using a transmission electron microscope (HITACHI, HT7800/HT7700), and images were captured for analysis.

Total ROS detection

Heart tissues (50–100 mg) were rapidly excised from euthanized B6j mice, rinsed in ice-cold PBS (0.01 M, pH 7.4) to remove residual blood, blotted dry, and homogenized on ice in 300 μL ROS assay buffer (S0033M; Beyotime, China) using a glass–Teflon homogenizer. Homogenates were centrifuged at 12,000 × g for 10 min at 4 °C, and the supernatants were collected for protein quantification by BCA assay. Samples were then normalized to 1 mg/mL total protein, and 100 μL aliquots were dispensed into black 96-well plates. A 10 μM working solution of the DCFH-DA probe was freshly prepared by diluting the 10 mM stock in assay buffer, and 100 μL was added per well. Following gentle mixing, plates were incubated in the dark at 37 °C for 30 min, washed twice with 200 μL PBS to remove unincorporated probe, and fluorescence was

measured at excitation/emission wavelengths of 485/530 nm on a microplate reader. Relative fluorescence units (RFU) were normalized to protein content (RFU/mg protein). Blank (buffer only) and positive control (50 μg/mL Rosup) wells were included to confirm assay performance.

Flow cytometry analysis

Hearts were collected on the third day following MI and analyzed via flow cytometry using a Gallios flow cytometer (Beckman Coulter Inc., GA, USA). The flow cytometry analysis was conducted using FlowJo software v10.8.0 (Tree Star Inc., OR, USA). The experiments were performed as previously described (Sokol L, Geldhof V, Garcia-Caballero M 2021).

The mice were anesthetized with 2–3% isoflurane and euthanized via cervical dislocation, followed by careful extraction of the heart for tissue collection. The hearts were placed in ice-cold medium and sectioned into 1 mm × 1 mm pieces under sterile conditions. The tissue was digested in a water bath at 37 °C for 20 minutes in a solution containing 0.5% (w/v) collagenase IV and 0.5 U/mL dispase in DMEM. Cell viability and counts were assessed using Trypan blue exclusion staining. The digestion was terminated by adding an equal volume of FACS buffer (2 mM EDTA and 0.5% BSA in D-PBS). The cell suspension was filtered through a 40 μm mesh, and the cell pellet was collected by centrifugation. The cells were resuspended in FACS buffer, stained with the Zombie Aqua™ Fixable Viability Kit (BioLegend) to assess viability, and incubated with fluorophore-conjugated antibodies (anti-CD86, anti-CD206, and anti-F4/80; all at a 1:100 dilution). The data were analyzed using FlowJo v10.8.0 software (Tree Star) with gating based on fluorescence minus one (FMO) and single-antibody controls. Doublet exclusion, debris exclusion, and viability gating were applied to isolate viable single-cell populations. F4/80, CD86, and CD206 gating were performed using FMO controls to quantify the $CD86^{+}CD206^{-}$ (M1-like macrophages) and $CD86^{-}CD206^{+}$ (M2-like macrophages) populations.

Statistical analysis

Continuous variables are presented as the means ± standard deviations. Statistical analyses were performed using ANOVA, independent-sample t tests, Mann–Whitney *U* tests, χ^2 tests, Pearson’s correlation analyses, and Spearman’s correlation analyses. Statistical significance was defined as *$p < 0.05$. The data were analyzed using PRISM 9.0 statistical software.

References

1. Gao E, Lei YH, Shang X, Huang ZM, Zuo L, Boucher M, et al. A novel and efficient model of coronary artery ligation and myocardial infarction in the mouse. Circ Res. 2010; 107(12):1445-1453.

2. Zhu F, Li Y, Zhang J, Piao C, Liu T, Li HH, et al. Senescent cardiac fibroblast is critical for cardiac fibrosis after myocardial infarction. PLoS One. 2013;

8(9):e74535.
3. Weyers JJ, Carlson DD, Murry CE, Schwartz SM, Mahoney WM, Jr. Retrograde perfusion and filling of mouse coronary vasculature as preparation for micro computed tomography imaging. J Vis Exp. 2012; (60):e3740.
4. Lu Y, Han G, Zhang Y, Zhang L, Li Z, Wang Q, et al. M2 macrophage-secreted exosomes promote metastasis and increase vascular permeability in hepatocellular carcinoma. Cell Commun Signal. 2023; 21(1):299.

2. Supplemental Tables

Table S1：Clinical baseline information table

	Overall（32）	MMI（23）	Control（9）	*p*-value
Age	63.81±12.53	62.83±11.424	66.33±15.476	0.486
Gender	13/19	10/13	3/6	0.900
Past medical history				
HBP	16/16	12/11	4/5	1.000
Hyperthyreosis	32/0	23/0	9/0	-
HDL	1.16±0.29	1.13±0.24	1.27±0.38	0.255
LDL	2.23±0.98	2.22±0.93	2.24±1.16	0.961
triacylglycerol	1.50±1.23	1.39 ±0.97	1.80±1.86	0.431
LVID	48.53±6.82	46.86±6.718	52.44±5.570	0.037*
FS	34.48±5.19	35.95±4.421	29.33±4.590	0.004*
EF	55.28±18.79	59.61±19.674	44.22±10.604	0.035*
WBC	5.95±1.73	5.6±1.60	6.82±1.83	0.074
CRP	15.6±24.18	20.89±29.54	6.45±5.12	0.368
PCT	0.16±0.64	0.22±0.76	0.03±0.06	0.480

Table S2：Drugs list

Compound ID	Compound name	signature strength	correlation coefficient	transcriptional activity score	connectivity score
BRD-K54416256	methimazole	112	0.2355	0.164223	-0.4448
BRD-K45152786	merbarone	283	0.4384	0.356171	-0.4447
BRD-K81672972	dinoprost	160	0.3816	0.249859	-0.4447
BRD-K48178389	BRD-K48178389	177	0.29	0.229095	-0.4447
BRD-A75409952	wortmannin	251	0.39	0.316373	-0.4446
BRD-	ST-4029573	268	0.32	0.296124	-0.4446

K16798053					
BRD-K58788895	BRD-K58788895	149	0.24	0.191218	-0.4446
BRD-A47598013	citalopram	250	0.31	0.281502	-0.4446
BRD-K69726595	mericitabine	266	0.648	0.419816	-0.4445
BRD-K78431006	crizotinib	284	0.3494	0.318531	-0.4444
BRD-K25944327	BRD-K25944327	154	0.3874	0.246985	-0.4445
BRD-K71799949	carbamazepine	133	0.37	0.224314	-0.4445
BRD-K01976263	emetine	147	0.33	0.222713	-0.4444
BRD-K57166447	BRD-K57166447	142	0.32	0.215551	-0.4444
BRD-K33453211	levocabastine	139	0.2629	0.193301	-0.4444
BRD-K63279176	BRD-K63279176	135	0.23	0.178181	-0.4443
BRD-K07310275	PF-04691502	542	0.69	0.618379	-0.4443
BRD-K03536150	BRD-K03536150	124	0.32	0.201426	-0.4443
BRD-K30677119	PP-30	305	0.5	0.39488	-0.4444
BRD-K51831558	BRD-K51831558	111	0.33	0.19353	-0.4444

BRD-K53592093	BRD-K53592093	363	0.36	0.36554	-0.4443
BRD-K68174511	torin-2	377	0.8077	0.55799	-0.4443
BRD-K73255294	nutlin-3	480	0.71	0.59031	-0.4443
BRD-K87737963	CYT-387	205	0.518	0.329513	-0.4443
BRD-K93971872	SA-84902	133	0.21	0.168992	-0.4442
BRD-K59469039	AG-879	379	0.6	0.482198	-0.4442
BRD-K92656786	BRD-K92656786	137	0.21	0.171514	-0.4441
BRD-K03406345	azacitidine	406	0.6986	0.538527	-0.4441
BRD-K67844266	MLN-4924	299	0.5027	0.392031	-0.4441
BRD-K49830011	BRD-K49830011	181	0.21	0.197142	-0.4441
BRD-A21406558	BRD-A21406558	317	0.42	0.368965	-0.4441
BRD-K71289571	zafirlukast	129	0.34	0.21177	-0.4441
BRD-K62342148	BRD-K62342148	102	0.27	0.167808	-0.444
BRD-K08799216	pelitinib	290	0.58	0.414709	-0.444
BRD-K55260	BRD-K55260239	121	0.21	0.161188	-0.444

239					
BRD-A23723433	paclitaxel	158	0.48	0.278471	-0.4439
BRD-K93830491	BRD-K93830491	217	0.55	0.349335	-0.444
BRD-K53669914	BRD-K53669914	139	0.2267	0.1795	-0.444
BRD-K98004573	rupatadine	336	0.3996	0.370521	-0.4439
BRD-A64485570	trifluridine	147	0.3805	0.239148	-0.4439
BRD-A82772293	mepazine	132	0.3012	0.201625	-0.4439
BRD-K57080016	selumetinib	224	0.36	0.287148	-0.4438
BRD-K26211296	BRD-K26211296	137	0.27	0.194479	-0.4438
BRD-K41859756	NVP-AUY922	335	0.61	0.457107	-0.4437
BRD-K51377689	BRD-K51377689	155	0.35	0.235521	-0.4437
BRD-K67868012	PI-103	408	0.62	0.508577	-0.4437
BRD-K05104363	PD-184352	709	0.8281	0.77481	-0.4437
BRD-K34014345	naproxol	106	0.21	0.150867	-0.4436
BRD-K28470988	L-690330	171	0.31	0.232814	-0.4436
BRD-	testosterone	172	0.23	0.201122	-0.4436

K90553655					
BRD-K64622987	torcetrapib	177	0.4744	0.293015	-0.4436
BRD-K46441700	GR-55562	125	0.21	0.163831	-0.4436
BRD-K41666683	BRD-K41666683	153	0.27	0.205522	-0.4436
BRD-K68158690	BRD-K68158690	140	0.23	0.181451	-0.4435
BRD-K55242822	BRD-K55242822	230	0.32	0.274328	-0.4435
BRD-K82469533	BRD-K82469533	241	0.36	0.297845	-0.4435
BRD-K06593056	LE-135	175	0.4392	0.280337	-0.4435
BRD-U33728988	QL-X-138	340	0.61	0.460506	-0.4435
BRD-A20527803	IB-MECA	140	0.3562	0.225809	-0.4434
BRD-K88069074	BRD-K88069074	126	0.29	0.193292	-0.4434
BRD-A80386041	KUC103423N	183	0.27	0.22477	-0.4433
BRD-K30351863	BRD-K30351863	359	0.5	0.428413	-0.4433
BRD-A44133049	azasetron	137	0.25	0.187138	-0.4433
BRD-K87158025	benzamil	111	0.33	0.19353	-0.4433

BRD-K08006444	BRD-K08006444	129	0.25	0.181591	-0.4432
BRD-K32744045	disulfiram	527	0.5	0.519064	-0.4432
BRD-K02292852	cefoperazone	139	0.2555	0.190561	-0.4432
BRD-K14767410	SC-560	121	0.23	0.168689	-0.4431
BRD-K20859359	BRD-K20859359	134	0.21	0.169626	-0.4431
BRD-K74514084	pazopanib	179	0.2112	0.196609	-0.4431
BRD-K24943235	grazoprevir	106	0.2206	0.154627	-0.4431
BRD-K16189898	CHIR-99021	293	0.45	0.367173	-0.4431
BRD-K46155530	BRD-K46155530	143	0.23	0.183384	-0.443
BRD-K35832492	BRD-K35832492	252	0.52	0.366043	-0.443
BRD-K68191783	ALW-II-38-3	389	0.47	0.432369	-0.443
BRD-K90072296	ML-4054	153	0.25	0.197764	-0.4429
BRD-K37142460	MI-2	502	0.77	0.628677	-0.4429
BRD-K82750814	BRD-K82750814	235	0.48	0.339614	-0.4429
BRD-K16730	regorafenib	469	0.66	0.562586	-0.4429

910					
BRD-K59730983	BRD-K59730983	111	0.33	0.19353	-0.4429
BRD-K09711437	varespladib	130	0.3815	0.22519	-0.4429
BRD-U44700465	HG-5-88-01	491	0.38	0.43678	-0.4429
BRD-K02265150	amoxapine	160	0.4295	0.265077	-0.4429
BRD-K62412498	BRD-K62412498	131	0.28	0.193662	-0.4428
BRD-K70914287	BIBX-1382	181	0.2692	0.223207	-0.4428
BRD-K05639119	SA-1920013	259	0.31	0.286524	-0.4428
BRD-K30007764	BRD-K30007764	208	0.27	0.239632	-0.4428
BRD-K67859584	BRD-K67859584	119	0.42	0.226063	-0.4428
BRD-K84157702	BRD-K84157702	155	0.21	0.182434	-0.4428
BRD-A18246003	monoethylhexylphthalate	310	0.39	0.351596	-0.4428
BRD-K44442813	pidotimod	109	0.2523	0.167688	-0.4428
BRD-K98109757	cyclopentene	202	0.37	0.276444	-0.4427
BRD-K48864121	BRD-K48864121	151	0.4894	0.274885	-0.4427
BRD-	BAY-11-7082	200	0.44	0.299966	-0.4427

K15025317					
BRD-K41430135	BRD-K41430135	110	0.4	0.212108	-0.4427
BRD-K31495718	AZD-7687	121	0.2765	0.184957	-0.4427
BRD-K77663706	BRD-K77663706	120	0.33	0.201223	-0.4427
BRD-K31109389	BRD-K31109389	194	0.43	0.292056	-0.4426
BRD-K49477212	BRD-K49477212	115	0.24	0.167991	-0.4426
BRD-K39256324	rottlerin	236	0.38	0.302816	-0.4426

Table S3：Main reagents list

**Reagent**	**Cat.**	**Manufacturer**
Anti-GAPDH	A19056	Abclonal
Anti-β-tubulin	ab6046	Abcam
Anti-TNF-α	sc-52746	Santacruz
Anti-IL-1β	12242s	CST
Anti-IL-6	12912S	CST
Anti-IL-10	sc-73309	Santacruz
Anti-p-ERK1/2	4370s	CST
Anti-VEGF	19003-1-AP	Proteintech
Anti-MAPK1	4695s	CST
Anti-T-ERK1/2	4695s	CST
Anti-CD31	AF3628	R&D System
Anti-ki67	ab16667	Abcam
Cy3-conjugated Anti-α-SMA	c6198	sigma
AF488-conjugated Anti-CD68	ab201844	Abcam
Anti-CD86	ab11985	Abcam

	7	
Anti-CD206	24595s	CST
Anti-F4/80	NB-600-404	Novus
APC anti-mouse F4/80 Recombinant Antibody	157305	Biolegend
CD86 (B7-2) Monoclonal Antibody (GL1), PE-Cyanine7, eBioscience™	25-0862-82	Thermo Fisher
Brilliant Violet 785™ anti-mouse CD206 (MMR) Antibody	141729	Biolegend
Zombie Aqua™ Fixable Viability Kit	423101	Biolegend
Honokiol	HY-N0003	MCE
Methimazole	HY-B0208	MCE
osmic acid	18456	Ted Pella Inc
812 embedding agent	90529-77-4	SPI
Uranyl acetate	02624-AB	SPI
Lead nitrate	203580	Sigma

Table S4：Primer list

Gene	Forward	Reverse
hsa-TNF-α	GAGGCCAAGCCCTGGTATG	CGGGCCGATTGATCTCAGC
hsa-IL-10	GACTTTAAGGGTTACCTGGGTTG	TCACATGCGCCTTGATGTCTG
hsa-IL-1β	ATGATGGCTTATTACAGTGGCAA	GTCGGAGATTCGTAGCTGGA
hsa-IL-6	ACTCACCTCTTCAGAACGAATTG	CCATCTTTGGAAGGTTCAGGTTG
hsa-VEGF	AGGGCAGAATCATCACGAAGT	AGGGTCTCGATTGGATGGCA
hsa-β-actin	CATGTACGTTGCTATCCAGGC	CTCCTTAATGTCACGCACGAT
hsa-GAPDH	ACAACTTTGGTATCGTGGAAGG	GCCATCACGCCACAGTTTC
mmu-TNF-α	CCCTCACACTCAGATCATCTTCT	GCTACGACGTGGGCTACAG
mmu-IL-10	GCTCTTACTGACTGGCATGAG	CGCAGCTCTAGGAGCATGTG

mmu-IL-1β	GCAACTGTTCCTGAACTCA ACT	ATCTTTTGGGGTCCGTCAA CT
mmu-IL-6	TAGTCCTTCCTACCCCAAT TTCC	TTGGTCCTTAGCCACTCCT TC
mmu-VEGF	GCACATAGAGAGAATGAGC TTCC	CTCCGCTCTGAACAAGGC T
mmu-β-actin	GGCTGTATTCCCCTCCATC G	CCAGTTGGTAACAATGCCA TGT
mmu-GAPDH	AGGTCGGTGTGAACGGAT TTG	TGTAGACCATGTAGTTGAG GTCA

3. Supplemental Figures

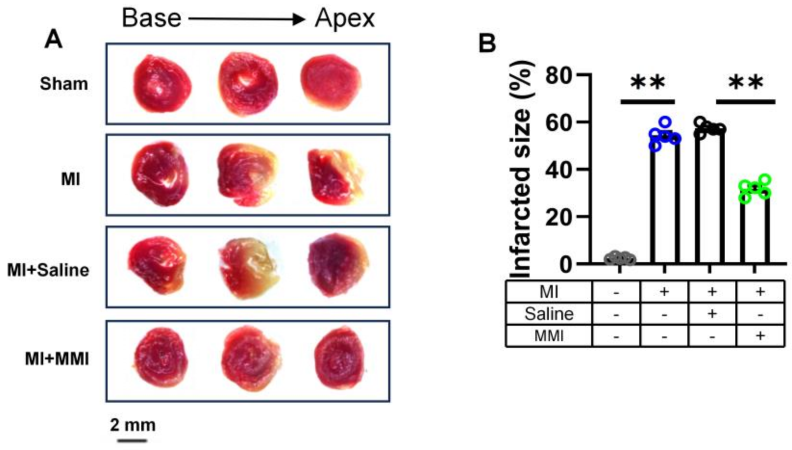

Figure S1. The effect of MMI on heart at early stage of MI

A and **B**. TTC staining image (A) and quantification (B) of B6j mice heart in sham or MI with saline or MMI treatment, scale bar, 2 mm, n = 5 mice for each group. One-way ANOVA with Tukey multiple comparisons test in (B) (**$p <$ 0.01). Each dot represents a single mouse. Data are represented as mean ± SEM.

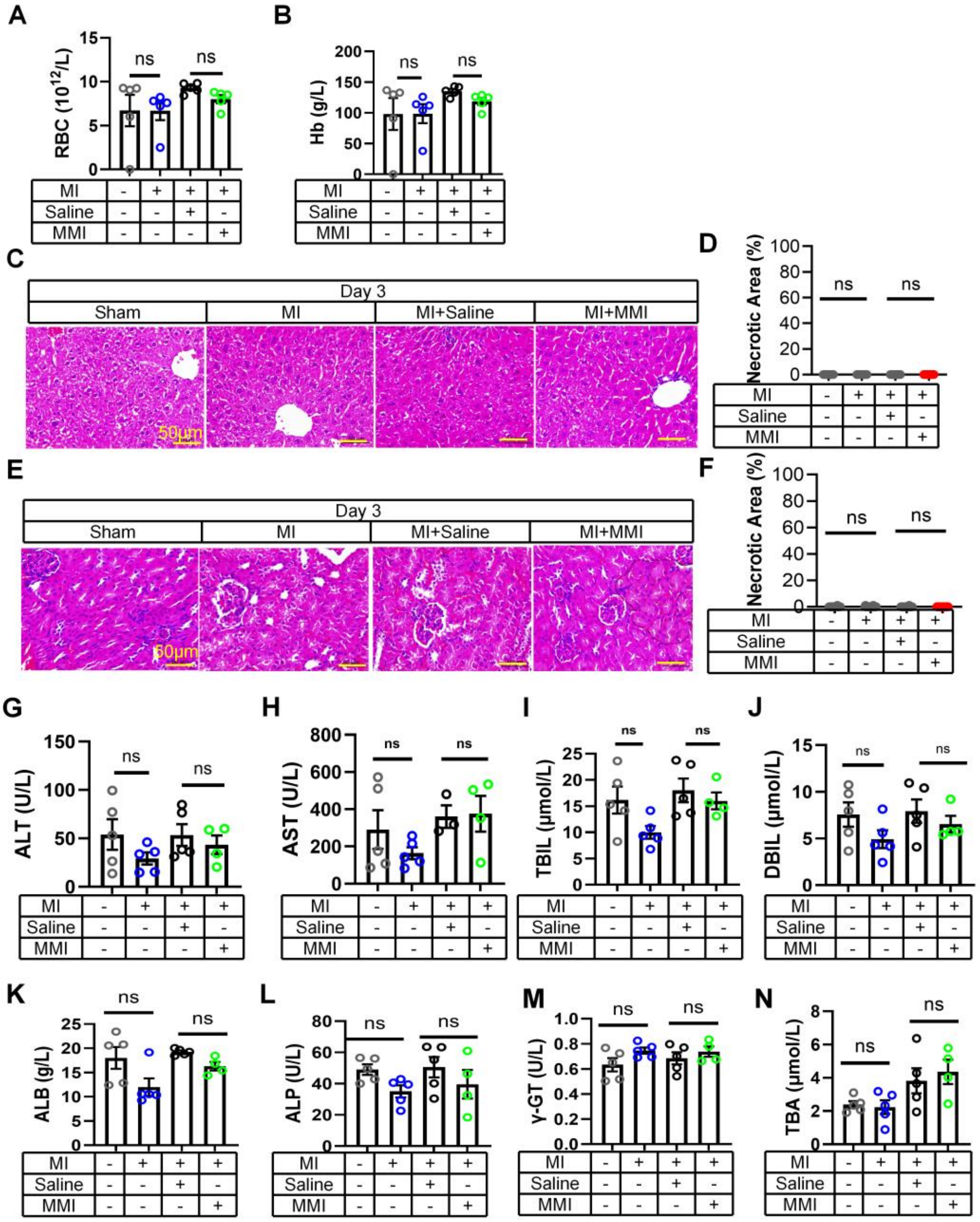

Figure S2. The effect of MMI on blood routine, liver and kidney function in mice

A and **B**. Quantification of red blood cells counts (A) and hemoglobin (B) from the serum of B6j mice in sham or MI with saline or MMI treatment, n = 5 mice for each group. **C** and **D**. Representative images of liver H&E staining (C) and quantification of necrotic area (D) from A at 3 days after MI in mice with application of MMI or saline, scale bar, 50 μm, n = 5 mice for each group. **E** and **F**. Representative images of renal H&E staining (E) and quantification of necrotic area (F) at day 3 after MI with application of MMI or saline, scale bar, 50 μm, n = 5 mice for each group. **G**-**N**. Quantification of ALT (G) and AST levels (H), TBIL (I), DBIL (J), ALB (K), ALP (L), γ-GT (M) and TBA (N) from the

serum of B6j mice in sham or MI with saline or MMI treatment following MI injury, n = 5 mice for each group. One-way ANOVA with Tukey multiple comparisons test in (A, B, D, F-N) (*$p < 0.05$, **$p < 0.01$, ns, not significant). Each dot represents a single mouse. Data are represented as mean ± SEM.

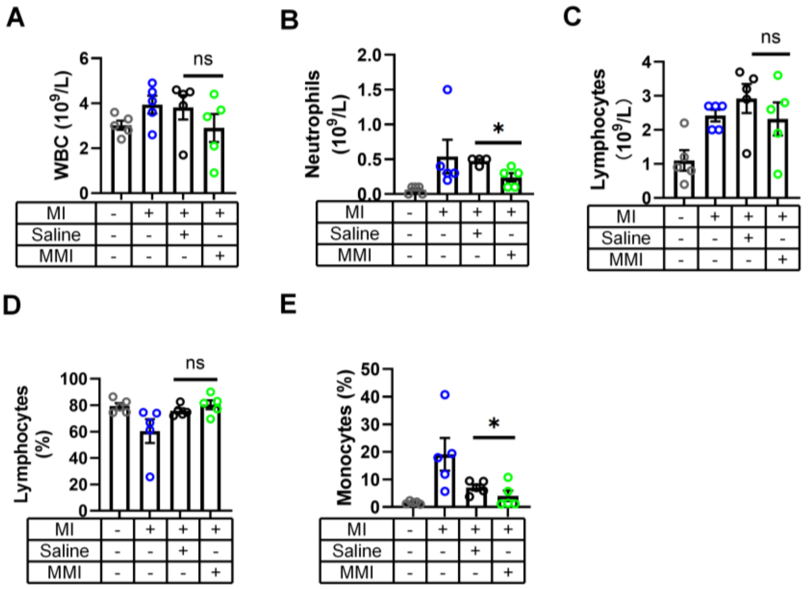

Figure S3. The effect of MMI on myeloid cell in murine MI model

A. Quantification of white blood cells counts from the serum of B6j mice in sham or MI with saline or MMI treatment, n = 5 mice for each group. **B.** Quantification of neutrophils ratio (%) from the serum of B6j mice in sham or MI with saline or MMI treatment, n = 5 mice for each group. **C** and **D**. Quantification of lymphocytes counts (C) and lymphocytes ratio (%) (D) from the serum of B6j mice in sham or MI with saline or MMI treatment, n = 5 mice for each group. **E.** Quantification of monocytes ratio (%) from the serum of B6j mice in sham or MI with corn oil or selumetinib treatment, n = 5 mice for each group. All the results of blood routine were derived from blood collected from hearts of mice, which were treated with MMI or saline at 3 days after MI. One-way ANOVA with Tukey multiple comparisons test (*$p < 0.05$, **$p < 0.01$, ns, not significant). Each dot represents a single mouse. Data are represented as mean ± SEM.

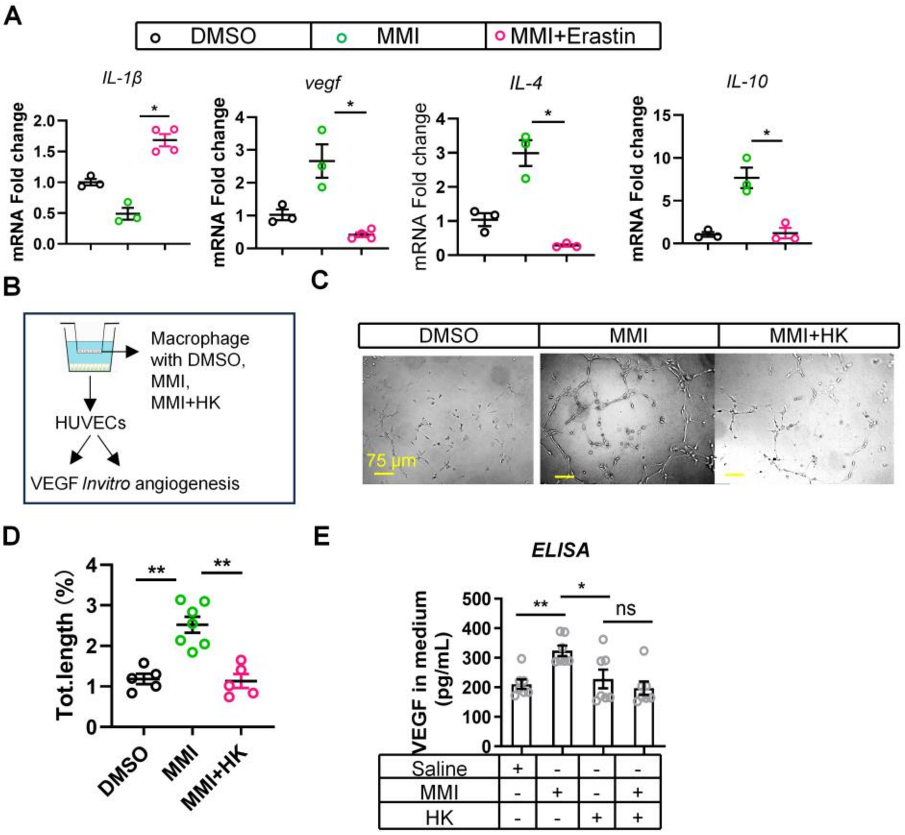

Figure S4. HK reversed MMI mediated pro-angiogenesis in macrophage and ECs coculture system

A. Quantitative RT-PCR analysis of IL-1β, IL-10, IL-4 and VEGF in macrophage administrated with MMI or MMI and Erastin. **B.** Schematic diagram of the co-cultured system of THP-1 cells derived macrophage and human umbilical vein endothelial cells (HUVECs). **C** and **D.** Representative images (C) and quantification (D) of *in vitro* angiogenesis in HUVEC after coculturing with THP-1 derived macrophage treated with DMSO, MMI with or without HK (scale bar, 75 μm). **E.** Quantification of VEGF level by ELISA in culture medium from THP-1 derived macrophages following DMSO, MMI and MMI with HK combination, n = 7 independent times. One-way ANOVA with Tukey multiple comparisons test (*$p < 0.05$, **$p < 0.01$, ns, not significant). Each dot represents a single mouse. Data are represented as mean ± SEM.

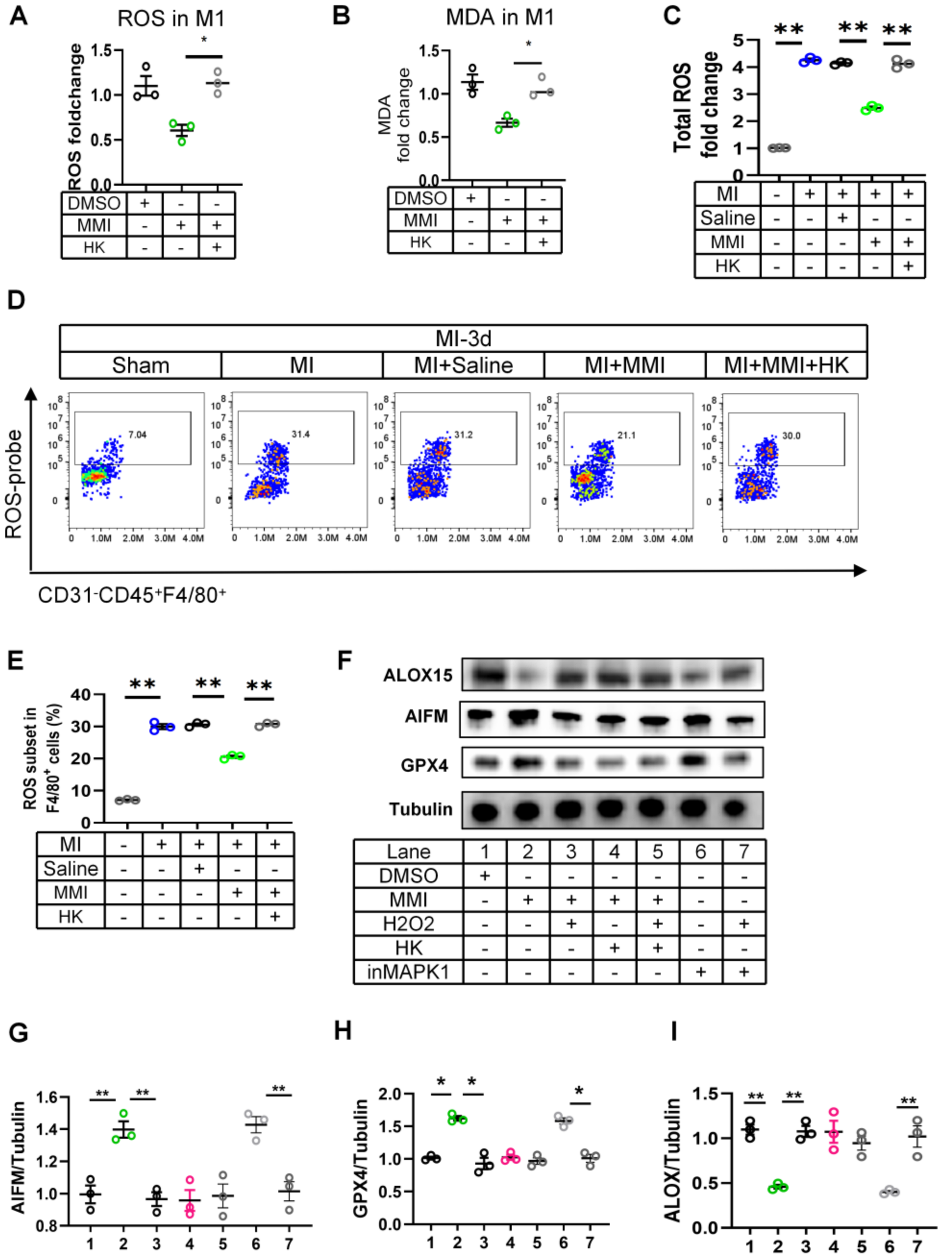

Figure S5. MAPK1 inhibition suppresses ferroptosis via attenuation of the ROS axis

A and **B**. Intracellular ROS (A) and MDA (B) levels were measured in M1-polarized macrophages treated with MMI alone or in combination with the HK, n = 3 independent experiment. **C**. Quantification of total cardiac ROS levels in B6j mice subjected to sham operation or MI and treated with saline, MMI, or

MMI + HK (n = 5 per group). **D–E**. Flow cytometric analysis of ROS in cardiac $F4/80^{+}$ $CD31^{-}CD45^{+}$ cells: representative plots (D) and corresponding quantification (E) from B6j mice in sham or MI groups treated with saline, MMI, or MMI + HK (n = 5 per group). **F**. Western blot analysis of ferroptosis-related proteins in M1 macrophages following the indicated treatments. **G–I**. Densitometric quantification of relative protein expression from (F): AIFM (G), GPX4 (H), and ALOX15 (I), n = 3 independent experiment. One-way ANOVA with Tukey multiple comparisons test in (A), (B), (C), (E), (G), (H) and (I) ($^{*}p < 0.05$, $^{**}p < 0.01$). Data are represented as mean ± SEM.

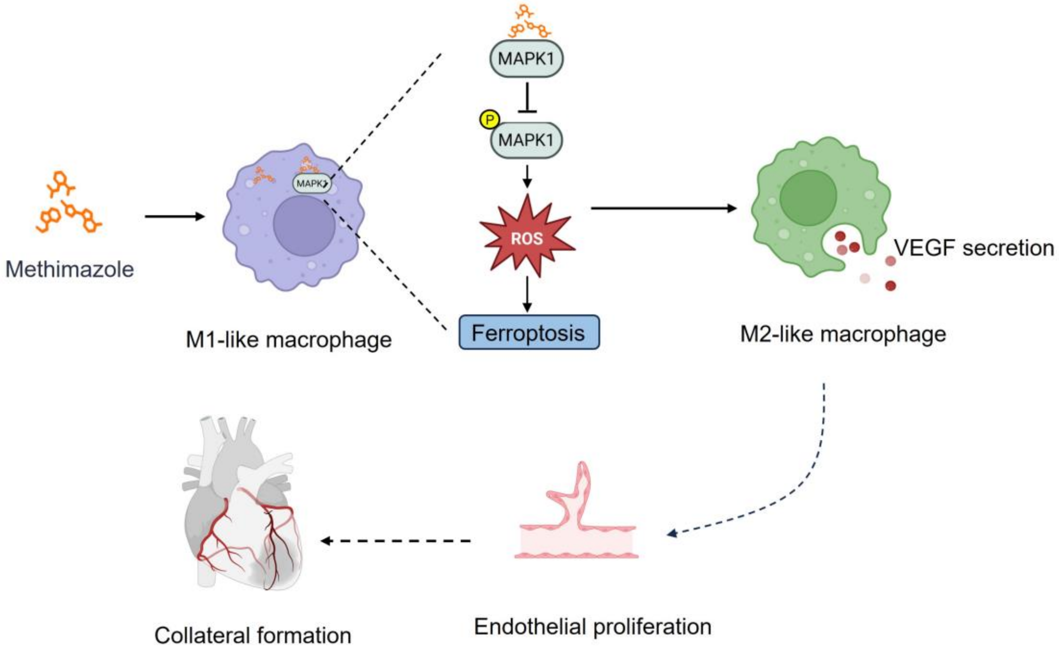

Figure S6. Model in our study

MMI binds to MAPK1 and inhibits its phosphorylation, thereby lowering ROS levels and protecting macrophages from ferroptosis. This ROS suppression drives their shift from an M1-like to an M2-like phenotype, ultimately increasing free VEGF availability and promoting coronary collateral formation after MI.
